# Effects of Angiotensin II Type 1A Receptor on ACE2, Neprilysin and KIM-1 in Two Kidney One Clip (2K1C) Model of Renovascular Hypertension

**DOI:** 10.3389/fphar.2020.602985

**Published:** 2021-01-29

**Authors:** Laale F. Alawi, Sanjeev Dhakal, Sana E. Emberesh, Harshal Sawant, Anhar Hosawi, Unmesha Thanekar, Nadja Grobe, Khalid M. Elased

**Affiliations:** Department of Pharmacology and Toxicology, Boonshoft School of Medicine, Wright State University, Dayton, OH, United States

**Keywords:** ACE2 (angiotensin converting Enzyme-2), neprilysin (NEP), hypertension, renin angiotensin aldestron system, chronic kidney, urinary bio markers, angiotensin II (A II) receptor antagonists, 2K1C Goldblatts’ model

## Abstract

Activation of the renin angiotensin system plays a pivotal role in the regulation of blood pressure, which is mainly attributed to the formation of angiotensin-II (Ang II). The actions of Ang II are mediated through binding to the Ang-II type 1 receptor (AT1R) which leads to increased blood pressure, fluid retention, and aldosterone secretion. In addition, Ang II is also involved in cell injury, vascular remodeling, and inflammation. The actions of Ang II could be antagonized by its conversion to the vasodilator peptide Ang (1–7), partly generated by the action of angiotensin converting enzyme 2 (ACE2) and/or neprilysin (NEP). Previous studies demonstrated increased urinary ACE2 shedding in the *db*/*db* mouse model of diabetic kidney disease. The aim of the study was to investigate whether renal and urinary ACE2 and NEP are altered in the 2K1C Goldblatt hypertensive mice. Since AT1R is highly expressed in the kidney, we also researched the effect of global deletion of AT1R on renal and urinary ACE2, NEP, and kidney injury marker (KIM-1). Hypertension and albuminuria were induced in AT1R knock out (AT1RKO) and WT mice by unilateral constriction of the renal artery of one kidney. The 24 h mean arterial blood pressure (MAP) was measured using radio-telemetry. Two weeks after 2K1C surgery, MAP and albuminuria were significantly increased in WT mice compared to AT1RKO mice. Results demonstrated a correlation between MAP and albuminuria. Unlike *db*/*db* diabetic mice, ACE2 and NEP expression and activities were significantly decreased in the clipped kidney of WT and AT1RKO compared with the contralateral kidney and sham control (*p* < 0.05). There was no detectable urinary ACE2 and NEP expression and activity in 2K1C mice. KIM-1 was significantly increased in the clipped kidney of WT and AT1KO (*p* < 0.05). Deletion of AT1R has no effect on the increased urinary KIM-1 excretion detected in 2K1C mice. In conclusion, renal injury in 2K1C Goldblatt mouse model is associated with loss of renal ACE2 and NEP expression and activity. Urinary KIM-1 could serve as an early indicator of acute kidney injury. Deletion of AT1R attenuates albuminuria and hypertension without affecting renal ACE2, NEP, and KIM-1 expression.

## Introduction

Chronic kidney disease (CKD) and end stage renal disease (ESRD) remain one of the major worldwide public health problems with increasing prevalence (Murphy et al., 2016). Hypertension and diabetes have been identified as the leading risk factors for the development of CKD and cardiovascular disease (Klag et al., 1996). In addition, uncontrolled hypertension is associated with high risk of cardiovascular morbidity and mortality. The two-kidney, one-clip (2K1C) Goldblatt hypertensive mouse model involves the constriction of renal arteries which leads to increased systolic blood pressure and development of renovascular hypertension (Goldblatt et al., 1934). The stenosis of the renal artery activates the renin angiotensin system (RAS) and is responsible for the upregulation of renin during the first few weeks (Prieto-Carrasquero et al., 2008; Grobe et al., 2015; Kim et al., 2016) which contributes to the upregulation of angiotensin II (Ang II) (Prieto et al., 2011). The Ang II-mediated hypertension through the AT1 receptor occurs during the entire period of renal artery stenosis regardless of the plasma renin activity (Wilcox et al., 1996). The 2K1C Goldblatt model is a useful tool to determine factors that might play a role in the development of cardiovascular and renal diseases in the absence of hyperglycemia and insulin resistance. Our previous study in the 2K1C Goldblatt hypertensive mice showed loss of the Ang-(1–7) forming enzyme prolyl carboxypeptidase which could impair the degradation of renal Ang II and contribute to kidney injury (Grobe et al., 2015). Recently, two other Ang-(1–7) forming enzymes, angiotensin converting enzyme 2 (ACE2) and neprilysin (NEP), have gained recognition as important regulators of cardiovascular and renal function. Besides playing a key role in the RAS, ACE2 has also been identified as the functional receptor for the severe acute respiratory syndrome (SARS-Cov) (Kuhn et al., 2004) and SARS-CoV-2, which causes the coronavirus disease 2019 (COVID-19) epidemic (Zhou et al., 2020). ACE2 plays a vital role in acute kidney disease and its inhibition was associated with increased inflammatory cytokines and infiltrates (Fang et al., 2013), and glomerular injury (Soler et al., 2007). One of the aims of the study was to investigate whether the renal injury seen in 2K1C Goldblatt hypertension increases urinary ACE2 and NEP shedding. ACE2 is highly expressed in several organs including the renal cortex (Ye et al., 2006) and has been shown to have a renoprotective function through the degradation of Ang II to Ang-(1–7) (Tipnis et al., 2000). ACE2 deficiency in mice is associated with the development of advanced glomerulosclerosis and acceleration of diabetic nephropathy (Oudit et al., 2006; Wong et al., 2007). There are reports of increased urinary ACE2 excretion in hypertensive and diabetic patients following treatment with the angiotensin receptor blocker olmesartan (Abe et al., 2015; Furuhashi et al., 2015). Therefore, in the present study, we attempted to induce renovascular hypertension and albuminuria in AT1KO mice to investigate the role of AT1R and hypertension on the Ang- (1–7) forming enzymes, ACE2 and NEP, and on the known biomarkers of acute kidney injury, KIM-1. The rationale of our hypothesis was based in part on our previous studies where there was increased shedding of ACE2 in the mouse model of diabetic kidney disease (Chodavarapu et al., 2013; Salem et al., 2014; Somineni et al., 2014). In the present study, urine samples from *db*/*db* were analyzed for ACE2 in parallel to 2K1C Goldblatt mouse model of renovascular hypertension.

Neutral endopeptidase (NEP) (EC 3.4.24.11) is a member of the neprilysin (M13) family of metallopeptidases. It activates the alternative pathway of the RAS and is considered to be a major contributor to the formation of Ang- (1–7) by cleaving Ang I (Yamamoto et al., 1992; Domenig et al., 2016). NEP is expressed in several organs, including the brain, heart, and lungs (Karoor et al., 2013), and is localized mainly in the proximal tubule of the kidney (Sakakibara et al., 1989). In addition, a soluble form of NEP is present and detectable in blood plasma, cerebrospinal fluid, amniotic fluid, and seminal plasma (Pavo et al., 2019). Elevated soluble NEP levels has been shown to be predictor of increased risk of recurrent all-cause, cardiovascular, and acute heart failure admissions in ambulatory patients with heart failure (Núñez et al., 2016). Previous studies in our laboratory have shown that decreased renal NEP in diabetic mice was restored with the management of glycemia by using pharmacological intervention or by physical exercise training (Elased et al., 2013; Alawi et al., 2020). The pharmacological inhibition of NEP with LCZ696 was associated with significant decrease in mortality of the heart failure patients (McMurray et al., 2014). However, the inhibition was associated with increased urinary albumin excretion, showing the physiological importance of NEP in the preservation of the kidney function (Voors et al., 2015). In addition, the increased urinary excretion of NEP in patients with CKD reflects the role of NEP as a biomarker for the detection of chronic kidney damage and also depicts the intrarenal NEP status (Pajenda et al., 2017). We thought to investigate the role of NEP in the 2K1C Goldblatt mouse model, since this subject has not been researched before.

The second aim of the study was to investigate whether deleting AT1R will attenuate renal and urinary kidney injury marker (KIM-1). Kidney injury molecule-1 (KIM-1), also known as T-cell immunoglobulin mucin 1, is a transmembrane glycoprotein originally discovered by Ichimura et al., in 1998 and has been shown to be upregulated in the proximal tubular cells following acute ischemic kidney injury (Ichimura et al., 1998). The ectodomain of KIM-1 is shed into the lumen, and serves as a urinary biomarker of human proximal tubule injury (Han et al., 2002).

2K1C Goldblatt animal model of renovascular hypertension has greatly contributed to our understanding of cardiovascular and renal diseases. The aim of the study was to test the hypothesis that urinary ACE2, NEP, and Kim-1 are elevated in this model and deletion of AT1R will attenuate the shedding of these candidates in the urine.

## Materials and Methods

### Animals

Male AT1a receptor knocked out (AT1KO) and their wild type control (AT1WT) mice were housed in a temperature -controlled environment at 22°C under a 12:12 h light: dark cycle with free access to water and standard mouse chow (Harlan Teklad, Madison, WI, United States). Mice were housed in group cages before surgery and housed individually postoperatively. Mice were originally provided by Dr Thomas Coffman (Duke University, Durham, NC, United States) and were generated from a breeding colony maintained at Wright State University (Dayton, OH, United States). Mice aged 12–16 weeks were used in the study and had freely access to standard mouse chow and water. Six-weeks old male *db/db* diabetic mice (BKS.Cg-Dock7*m* +/+ Lepr^*db*^/J) and their age-matched non-diabetic lean control mice (*db/m*) were obtained from Jackson Laboratories (Bar Harbor, ME, United States). All the experiments and surgical procedures carried out on the animals were approved by Wright State University Animal Care and Use Committee. All efforts we made to minimize pain and stress.

### Blood Pressure Measurement Using Telemetry Transmitter Implantation

Radio-telemetry probes were used to measure blood pressure in freely moving mice as described before (Grobe et al., 2015). Briefly, under isoflurane anesthesia, an incision was made on the neck and a sterile catheter of the telemetry transducer/transmitter (model TA11PA-C10, Data Sciences International, St Paul, MN, United States) was inserted into the carotid artery and secured with 5.0-gauge silk suture (Arosurgical, Newport Beach, CA, United States). The body of the transmitter device was placed in a subcutaneous sack on the animal’s flank. A suture was used to close the skin and the wound was cleaned with betaine solution. Subcutaneous Carprofen (5 mg/kg, Sigma-Aldrich, St. Louis, MO, United States) was administered immediately after surgery, and 24 h later for post-operative analgesia. Blood pressure and locomotor activity were measured 1 week after telemetry surgery, 2 days before the 2K1C surgery, second, and three weeks after 2K1C surgery.

### Goldblatt Two-Kidney, One Clip (2K1C) Surgery

Goldblatt 2K1C hypertension model was induced in WT and AT1KO mice as previously described (Grobe et al., 2015). Mice were randomly divided into four groups: WT (sham), WT (2K1C), AT1KO (sham) and AT1KO (2K1C) for the study. Under isoflurane anesthesia, a retro abdominal incision was made in the left flank and the left kidney was exposed. Then, a U-shaped sterile stainless-steel clip (Exidel SA, Delemont, Switzerland) was placed around the renal artery of the left kidney leaving a 0.12 mm gap for blood flow. The abdominal wall layer was sutured after the kidney was placed back in the retroperitoneal cavity. Carprofen (5 mg/kg, subcutaneously) was administered immediately after surgery, and 24 h later for post-operative analgesia. The same procedure was carried out in the sham groups without the insertion of the renal artery clip.

### Urinary Collection and Measurement of Creatinine and Albumin

For collection of 24-h urine samples, mice were placed individually in Tecniplast metabolic cages (West Chester, PA, United States) with free access to food and water. Urine samples were collected 1 week before surgery and weekly after placement of the renal artery clips. Urine samples were collected in presence of protease inhibitor cocktail containing 10 mM PMSF (Roche Diagnostics, Indianapolis, IN, United States) and centrifuged at 1,000 x g for 5 min at 4°C and the supernatants were stored at −80°C for later measurements. Urinary albumin was measured using an ELISA kit purchased from Bethyl Laboratories (Montgomery, TX, United States) as described before (Chodavarapu et al., 2013; Somineni et al., 2014). Urine samples were diluted with sample/conjugate buffer in the ratio 1:500. Assay was performed as per the instructions provided in the kit. Final absorbance was read at 450 nm using Fusion Packard plate reader (Packard BioScience, Meriden, CT, United States). Urinary creatinine levels were measured using an assay kit purchased from Quidel Corporation (San Diego, CA, United States) as described before (Alawi et al., 2020). Briefly, urine samples and standards were diluted to 1:40 and 25 µl of this diluted samples and standards were added to 96 well plate followed by 75 µl of colored solution and incubated at RT for 30 min. The reaction was stopped using 2 N H_2_SO_4_ and final absorbance was read at 490 nm using Fusion Packard plate reader (Packard BioScience, Meriden, CT, United States).

### Kidney Histology and Immunohistochemistry

Three weeks following Goldblatt 2K1C surgery, mice were deeply anesthetized with an intraperitoneal overdose of Euthasol (1 μg/g body weight). Transcardial perfusion was performed using cold 4% paraformaldehyde (in PBS, pH 7.5) and after fixation, kidneys were excised and post fixed in 4% paraformaldehyde at 4°C followed by dehydration and then embedded in paraffin. Paraffin-embedded kidney sections (5 μm) were processed and stained with periodic acid Schiff (PAS) and Masson’s trichome at AML laboratories (Baltimore, MD, United States) and examined under light microscope. For immunofluorescence, kidney sections were deparaffinizing, dehydrated with graded concentrations of ethanol and incubated with 10 mM sodium citrate buffer in water bath (95–99°C) for 30 min. Then, the kidney sections were permeabilized with 0.25% Triton in PBS, and blocked with 3% normal donkey serum diluted with 1X PBS 4°C for an hour. Sections were then incubated overnight at 4°C with diluted primary antibodies as follows: Goat anti-NEP (1:500, Cat # AF1126, R and D Systems, Minneapolis, MN, United States), goat anti-KIM-1 (1:500, Cat # AF 1817, R and D Systems, Minneapolis, MN, United States) and rabbit anti-ACE2 (1:500, Cat # HPA000288, Sigma, St. Louis, MO, United States). Then incubated with CY3 conjugated donkey anti-goat secondary antibody (1:500, code # 705-165-003, Jackson Immunoresearch, West Grove, PA, United States), Alex Fluor-conjugated 488 donkey anti-goat (1:500, Cat # A11055, Thermo Fisher Scientific, Waltham, MA, United States). Sections were mounted using a vectashield-mounting medium (Vector, Burlingame, CA). Images were acquired using a fluorescence microscope (Optronics, Goleta, CA, United States) and quantified using MetaMorph software (Molecular Devices, CA, United States).

### Western Blot

Three weeks following Goldblatt 2K1C surgery, animals were sacrificed, kidneys were collected, washed with complete Lysis buffer (Roche Diagnostics, Mannheim, Germany) and homogenized using a Precellys 24 homogenizer (Bertin technologies Montigny-le-Bretonneux, France) with 1.4 mm Zirconium oxide beads (Cayman Chemicals, Cat#10402) in complete lysis M-EDTA free buffer supplemented with 10 mM PMSF. Kidney homogenates were centrifuged at 10,000 × g in 4°C for 10 min to separate the cellular debris and the supernatants were collected for later use. Total protein was determined using Biorad reagent (Biorad, Hercules, CA, United States) and bovine serum albumin as a standard. Kidney lysates containing the loading buffer (Laemmli Sample Buffer +5% β mercaptoethanol, Bio-Rad, Hercules, CA) in 1:1 ratio was loaded in 8% SDS PAGE gel. The proteins from the gel were transferred into a 0.2 μm PVDF membrane (Millipore, Billerica, MA, United States). The membranes were blocked with 5% fat-free milk solution dissolved in PBS and Tween-20, and incubated with specific primary antibody goat anti-ACE2 (dilution in 1:1,000, R&D Systems, MN, United States), goat anti-Albumin (1:500, Santa Cruz, CA, United States), or goat anti-NEP (dilution in 1:2000, R&D system, MN, United States). The membranes were then incubated with secondary antibody donkey anti-goat (dilution in 1:2000, R&D system, United States), or donkey anti-rabbit (dilution in 1:20,000, Jackson Immunoresearch, United States). Super-signal chemiluminescent HRP substrate (Thermo Scientific, IL, United States) was used for the detection of the protein bands followed by exposing the membranes to autoradiography films and visualized using Medical film processor (Konica Minolta and Grac, Inc., Taiwan).

### Angiotensin Converting Enzyme (ACE2) Activity

Renal and urinary ACE2 activity were measured using the specific fluorogenic ACE2 substrate, Mca-APK-(Dnp) (Biomol International, NY, United States) in the presence and absence of the ACE2 inhibitor, MLN-460 (gift from Millennium Pharmaceuticals, Cambridge, MA) as described before with some modifications (Chodavarapu et al., 2013; Gutta et al., 2018). Diluted urine sample equivalent to 0.2 µg of creatinine and kidney lysates containing 1 µg of protein were pre incubated with 20 µl of assay buffer (50 mM Tris, 5 mM ZnCl_2_, 150 mM NaCl_2_ and 10 µM lisinopril) with or without MLN 4760 (10 μM) for 30 min at room temperature. After the incubation, 100 μL of the assay buffer containing the ACE2 substrate Mca-APK (Dnp) (50 μM) was added the reaction mixture and incubated for 15–60 min at room temperature. Florescence was measured at excitation (λ ex): 335 nm and emission (λ _em_): 405 nm using Biotek Synergy H1 plate reader (BioTek instruments, Winooski, VT, United States).

### Neprilysin (NEP) Activity

Renal and urinary NEP activity were measured using an indirect fluorogenic assay as described before (Alawi et al., 2020) with some modifications. The cleavage of the fluorogenic substrate, Succinyl-Ala-Ala-Phe-7-amido-4-methylcoumarin (Suc-Ala-Ala-Phe-AMC) (Sigma-Aldrich, St. Louis, MO, United States) by NEP generates hydrophobic residue (phenyalanine-methylcouramin). For each reaction, kidney lysates (0.75 μg protein) or urine samples equivalent (1 µg of creatinine) were incubated with assay buffer (50 mM Tris, 5 mM ZnCl_2_, 150 mM NaCl2 and 10 µM lisinopril) in the presence and absence of the NEP inhibitor, thiophen (10 μM), in black-colored 96-well plate (Corning Inc.) for 15 min. Then 100 μL of the reaction substrate (4 mM, Suc-Ala-Ala-Phe-AMC) was added to each well and the plate was incubated for 1 h at 37°C. After cleavage of the NEP substrate, 5 µl of 4 mM leucine aminopeptidase was added to each well followed by 2 µl of 1 mM phosphoramidon which liberates the fluorescent molecule AMC. The fluorescence was measured at excitation (λ ex): 390 nm and emission (λ _em_): 460 nm using Biotek Synergy H1 plate reader (Winooski, VT, United States).

## Result

### Early Onset of Renovascular Hypertension Was Attenuated in AT1KO Mice

**FIGURE 1:**
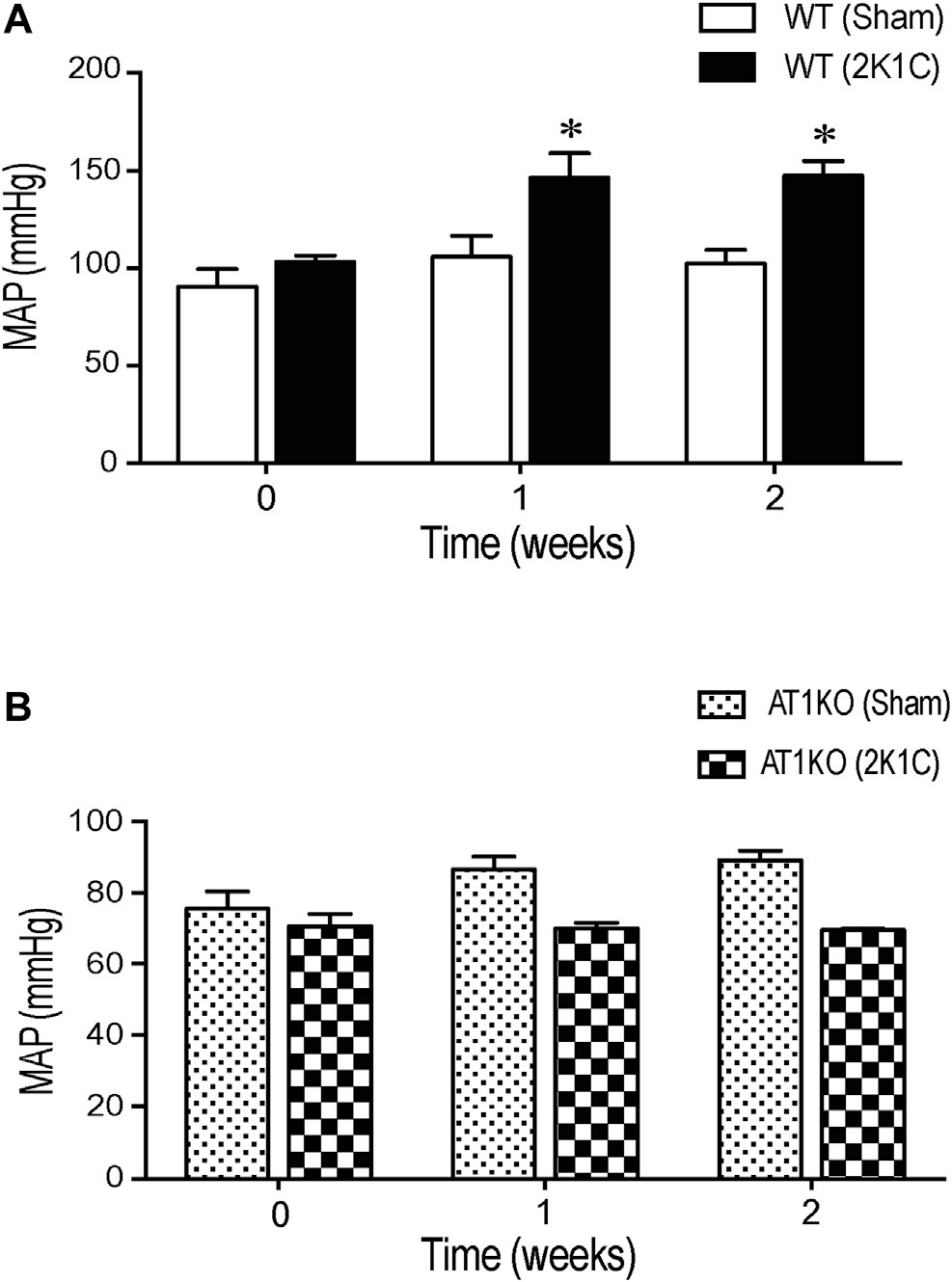
Effect of 2K1C Goldblatt surgery on 24 h mean arterial blood pressure in angiotensin-II (Ang-II) type 1 A receptors wild type (WT) and angiotensin-II (Ang-II) type 1 A receptors knock out (AT1KO) mice. Mean arterial pressure was measured at base line (0 weeks), 1 and 2 weeks following 2K1C surgery in **(A)** WT (sham) and WT (2K1C) **(B)** AT1KO (sham) and AT1KO (2K1C). ANOVA showed a significant increase of MAP in WT 2K1C mice compared to baseline (0 weeks) and WT sham mice during the 2 weeks of the study (*p < 0.05 vs. Sham). Data are represented as mean ± SEM, (n = 6–8).

Renovascular hypertension was induced in AT1KO and WT mice by placing renal clips in the left renal artery. After 1-week recovery, blood pressure was measured every other day during the 3 weeks of the study. There was a significant increase in MAP after renal artery clipping in WT 2K1C (147.3 ± 7.4 mm Hg) compared to age-matched WT sham-operated mice (102.3 ± 6.9 mm Hg) (Figure 1A, *p* < 0.05). Interestingly, in AT1KO mice, there was no significant difference in MAP at baseline (89.1 ± 2.6 mm Hg) and 2 weeks after renal artery clipping (70.5 ± 3.4 mm Hg) (Figure 1B). Moreover, there was no significant difference in MAP after renal artery clipping during the 2 weeks in AT1KO 2K1C compared to age-matched WT sham-operated mice (Figure 1B). As expected, MAP was significantly decreased in AT1KO mice compared with WT mice at baseline (75.4 ± 3.5 vs*.* 102 ± 6.9, Figure 1, *p* < 0.05). In summary, blood pressure measurements revealed a significant increase of 46.1 ± 3.6 mm Hg in 2K1C WT animals compared to age-matched WT sham-operated mice (*p* < 0.05). However, 2K1C surgery had no effect on MAP in AT1KO mice.

### Increased Urinary Albumin Excretion in 2K1C Mice Was Attenuated in AT1KO Mice

**FIGURE 2:**
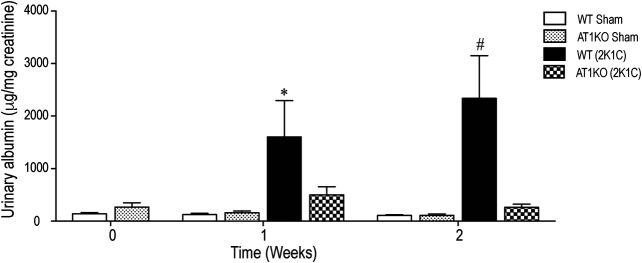
Effect of renovascular hypertension on urinary albumin excretion in WT and AT1KO mice after one and 2 weeks of 2K1C Goldblatt surgery. Urinary Albumin excretion was expressed as μg/creatinine in WT (sham), AT1KO (sham), WT (2K1C), and AT1KO (2K1C). One-way ANOVA showed a significant increase of albumin excretion in WT 2K1C mice after 1 week (*p < 0.001 vs. sham) and 2 weeks by (#*p* < 0.0001) compared to baseline, sham groups, and AT1 KO 2K1C mice. Data are represented as mean ± SEM, (n = 6–10).

Figure 2 shows the urinary albumin excretion in WT and AT1KO mice at baseline (0 weeks), one and 2 weeks following the 2K1C Goldblatt surgery. One week following 2K1C Goldblatt surgery in WT mice, urinary albumin excretion level was significantly increased (*p* < 0.001), and increased even more at the end of the second week compared with baseline and sham-operated mice (Figure 2, *p* < 0.0001). As expected, AT1KO mice showed no significant difference in urinary albumin excretion after one and two weeks of 2K1C surgery. In addition, the there was no significant difference in urinary albumin excretion in sham-operated mice for both WT and AT1KO mice compared to base lines at time 0 weeks (Figure 2).

### Decreased Renal ACE2 Protein Expression in Clipped Kidneys

**FIGURE 3:**
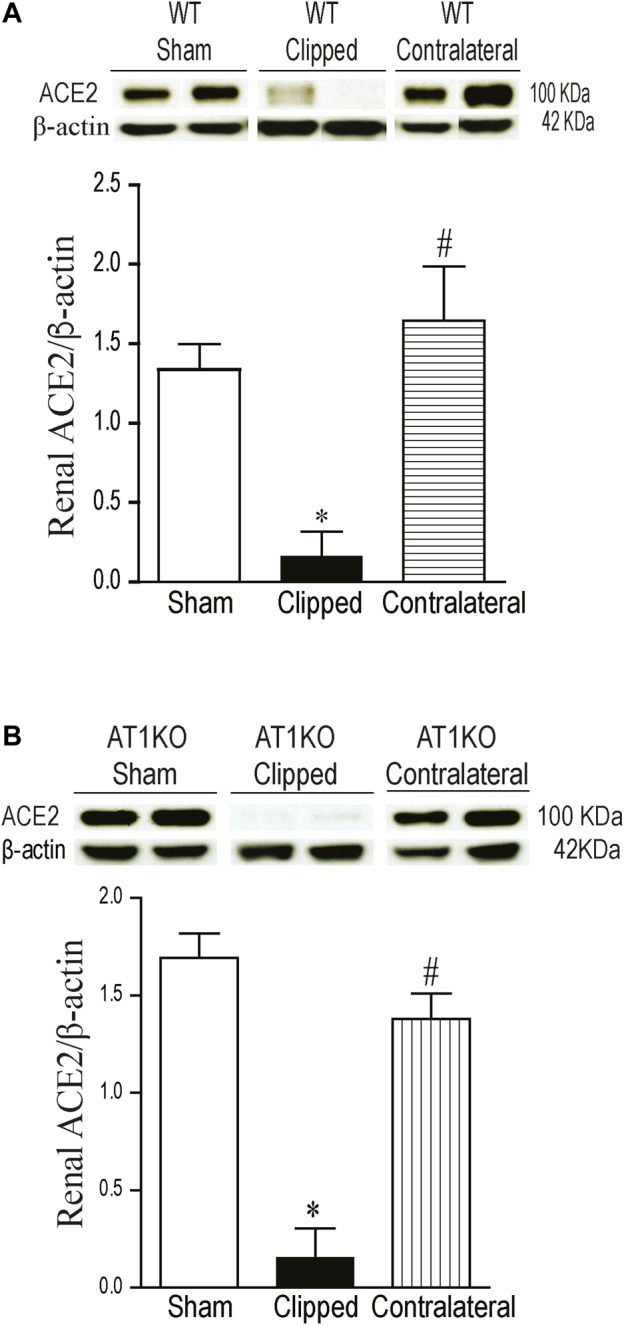
Effect of renovascular hypertension on renal ACE2 expression in WT and AT1KO mice after 2 weeks of 2K1C Goldblatt surgery. **(A)** Renal ACE2 protein expression in WT sham, clipped and contralateral kidney. **(B)** Renal ACE2 protein expression in AT1KO sham, clipped and contralateral kidney. In each individual experiment, samples were loaded randomly in four gels and processed at the same time. Two Immunoreactive bands corresponding to immunoreactive ACE2 from each experiment were appropriately cropped and spliced as a representative of the result. One-way ANOVA showed a significant decreased in renal ACE2 expression in WT and AT1KO clipped kidneys compared to sham and contralateral kidneys (*p < 0.001 vs. sham, #*p* < 0.001 vs. clipped). Data are represented as mean ± SEM, (n = 6).

A single immunoreactive band for renal ACE2 was detected at a molecular weight ∼100 KDa. This result is consistent with the full length of ACE2 as reported by previous studies (Chodavarapu et al., 2013; Somineni et al., 2014). In WT mice, renal expression of ACE2 was significantly decreased in the clipped kidneys compared to the sham-operated and contralateral kidneys (Figure 3A, *p* < 0.001). However, there was no significant difference in renal ACE2 between contralateral kidney and sham-operated controls. In AT1KO mice, renal ACE2 was also significantly decreased in the clipped kidneys compared to the sham-operated and contralateral AT1KO kidneys (Figure 3B, *p* < 0.001).

### Decreased Renal NEP Protein Expression in Clipped Kidneys

**FIGURE 4:**
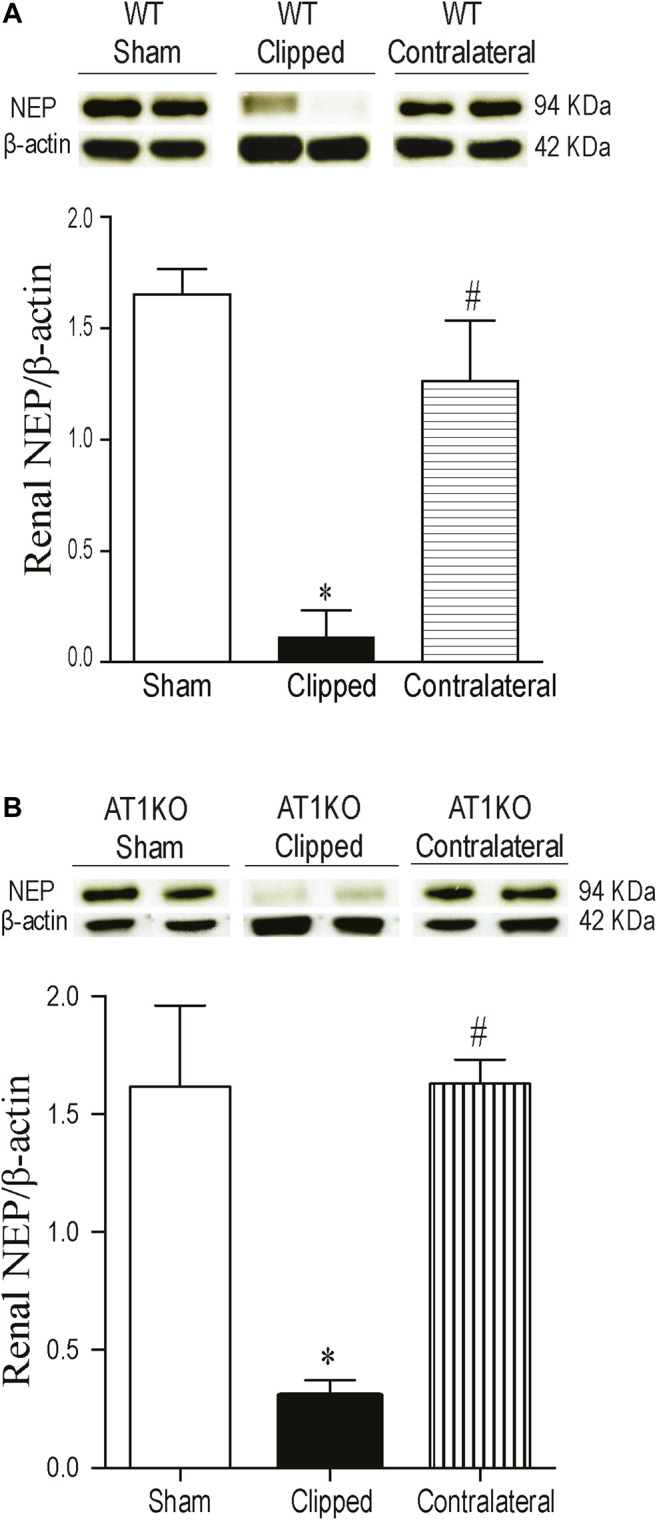
Effect of renovascular hypertension on renal NEP expression in WT and AT1KO mice after 2 weeks of 2K1C Goldblatt surgery. **(A)** Renal NEP protein expression in WT sham, clipped and contralateral kidney. **(B)** Renal NEP protein expression in AT1KO sham, clipped and contralateral kidney. In each individual experiment, samples were loaded randomly in four gels. Two Immunoreactive bands from each group corresponding to NEP from this experiment were appropriately cropped and spliced as a representative of the result. One-way ANOVA showed a significant decreased in renal NEP expression in WT and AT1KO clipped kidneys compared to sham and contralateral kidneys (*p < 0.001 vs. sham, #*p* < 0.001 vs. clipped). Data are represented as mean ± SEM, (n = 6).

A single immunoreactive band for renal NEP was detected at a molecular weight ≈ 95 KDa in clipped, contralateral, and sham-operated kidneys (Figure 4). The renal expression of NEP was significantly decreased in the clipped kidney compared with sham-operated controls and the contralateral WT kidneys (Figure 4A, *p* < 0.001). However, there was no difference in NEP protein expression between contralateral kidney and the sham-operated controls. In AT1KO mice, renal NEP was also significantly decreased in the clipped kidneys compared with the sham-operated control and contralateral kidneys (Figure 4B, *p* < 0.001).

### Increased Renal Renin Protein Expression in Clipped Kidneys

**FIGURE 5:**
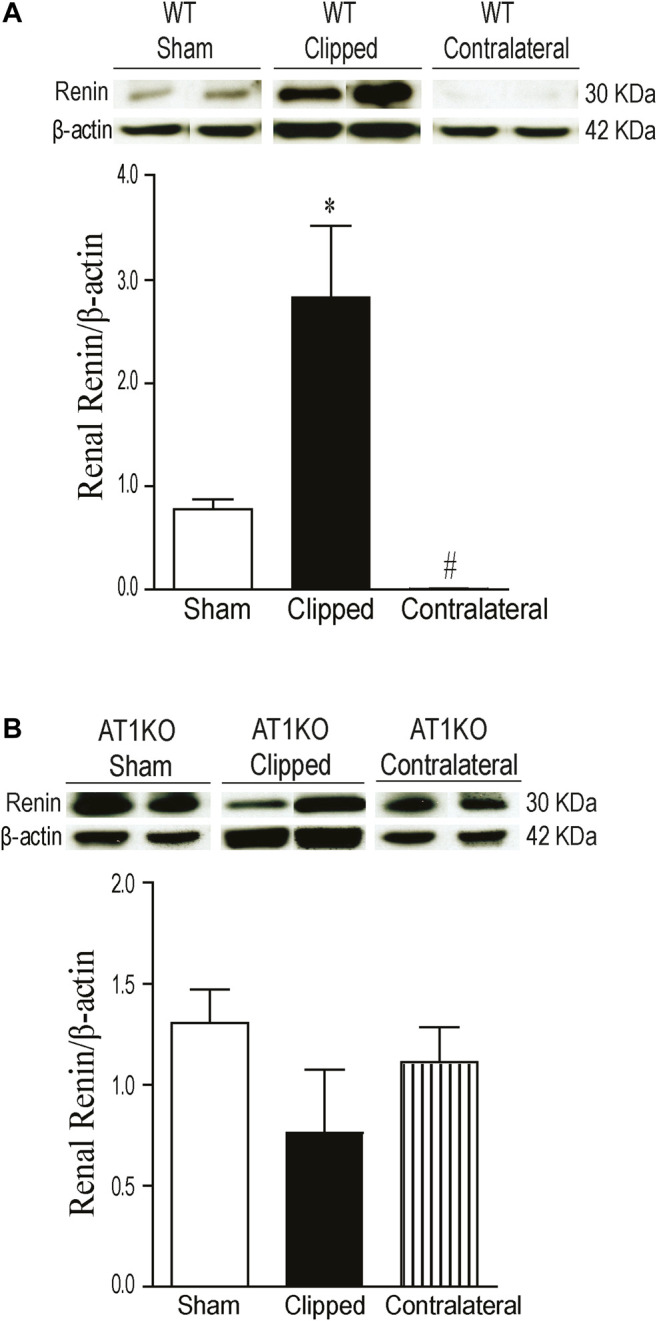
Effect of renovascular hypertension on renal renin expression in WT and AT1KO mice after 2 weeks of 2K1C Goldblatt surgery. (**A)** Renal renin protein expression in WT sham, clipped kidney and contralateral kidney. (**B)** Renal renin protein expression in AT1KO sham, clipped and contralateral kidney. In each individual experiment, samples were loaded randomly in four gels. Two Immunoreactive bands from each group corresponding to renin were appropriately cropped and spliced as a representative of the result. One-way ANOVA showed a significant increase of renal renin expression in WT clipped kidney of 2K1C mice compared to sham and contralateral kidney (*p < 0.001 vs. sham). Data are represented as mean ± SEM (n = 6).

The expression of renal renin was observed at a molecular weight ≈30 KDa in clipped, contralateral, and sham-operated kidneys (Figure 5). The renal expression of renin was significantly increased in the clipped kidney compared with the contralateral and sham-operated control (Figure 5A, *p* < 0.001). In addition, the expression of renal renin was significantly decreased in the contralateral kidney compared to the sham-operated control (Figure 5A, *p* < 0.001). However, in AT1KO mice, there was no significant difference in renal renin between the three groups (Figure 5B).

### Increased Renal KIM-1 Protein Expression in Clipped Kidneys

**FIGURE 6:**
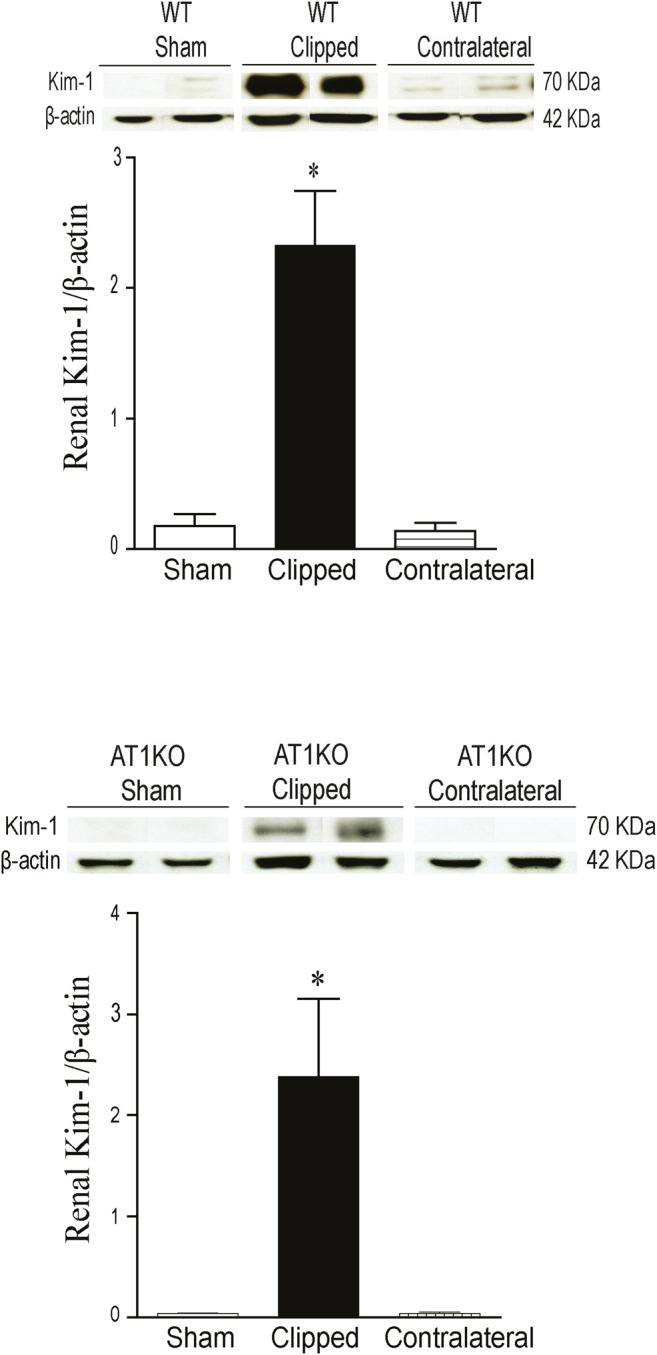
Effect of renovascular hypertension on renal KIM-1 expression in WT and AT1KO mice after 2 weeks of 2K1C Goldblatt surgery. **(A)** Renal KIM-1 protein expression in WT sham, clipped kidney and contralateral kidney. **(B)** Renal KIM-1 protein expression in AT1KO sham, clipped kidney and contralateral kidney. In each individual experiment, samples were loaded randomly in four gels. Two Immunoreactive bands from each group corresponding to KIM-1were appropriately cropped and spliced as a representative of the result. One-way ANOVA showed a significant increase of renal KIM-1 expression in WT and AT1KO clipped kidney compared to sham and contralateral (*p < 0.001 vs. Sham). Data are represented as mean ± SEM, (n = 6).

As expected, in the WT and AT1KO sham-operated kidneys there was almost no detectable protein expression of KIM-1 (Figure 6). The immunoreactive bands for renal KIM-1 were observed at a molecular weight of ≈ 70 KDa predominantly in the clipped kidneys of both WT and AT1KO mice and they were significantly increased compared with the faint bands in the sham-operated controls and contralateral kidneys (Figure 6, *p* < 0.001).

### Decreased Renal ACE2 and NEP Activity in Clipped Kidneys

**FIGURE 7:**
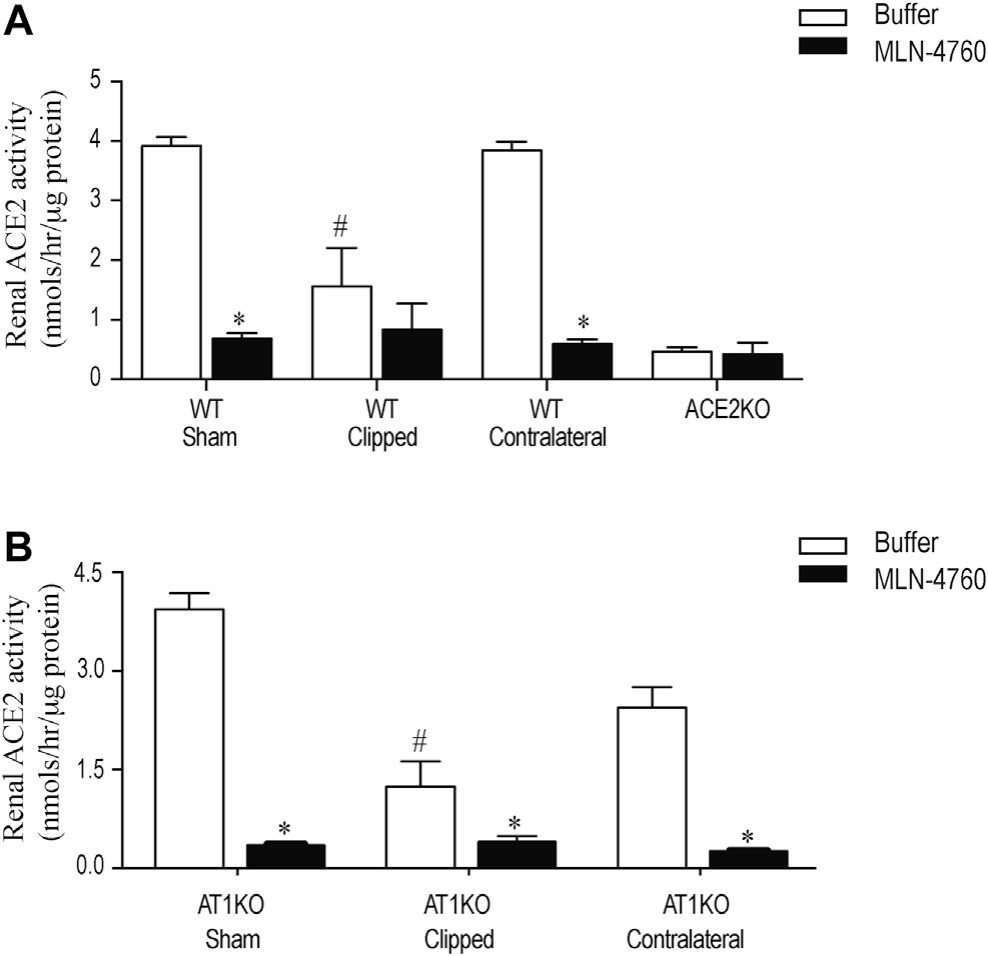
Effect of renovascular hypertension on renal ACE2 activity in WT and AT1KO mice after 2 weeks of 2K1C Goldblatt surgery. Renal ACE2 activity was measured using 20 µg of kidney protein and fluorogenic ACE2 substrate in presence and absence of the ACE2 inhibitor MLN-4760. **(A)** ACE2 activity in the sham, clipped and contralateral kidneys of WT mice. **(B)** ACE2 activity in the sham, clipped and contralateral kidneys of AT1KO mice. One-way ANOVA showed a significant decreased in renal ACE2 activity in clipped kidney of 2K1C mice compared to sham and contralateral kidney *p < 0.001 vs. buffer, #*p* < 0.05 vs. sham kidney. Each bar represents mean ± SEM (n = 6–8).

Renal ACE2 activity was measured in kidney lysates using the fluorogenic substrate, Mca-APK (Dnp) in the presence and absence of the ACE2 inhibitor, MLN-460 as described before (Alawi et al., 2020). In addition, the assay was validated by using kidney lysates from ACE2KO mice as a negative control (Figure 7A). The difference of fluorescence generated by kidney lysate in presence of buffer and MLN-460 is considered as ACE2 activity. Figure 7 shows a significant decreased of renal ACE2 activity in the WT and AT1KO clipped kidney compared with the contralateral kidneys and sham-operated controls (Figure 7, *p* < 0.001). There was no significant difference in ACE2 activity between the WT and AT1KO contralateral kidneys and the sham-operated controls (Figure 7).

**FIGURE 8:**
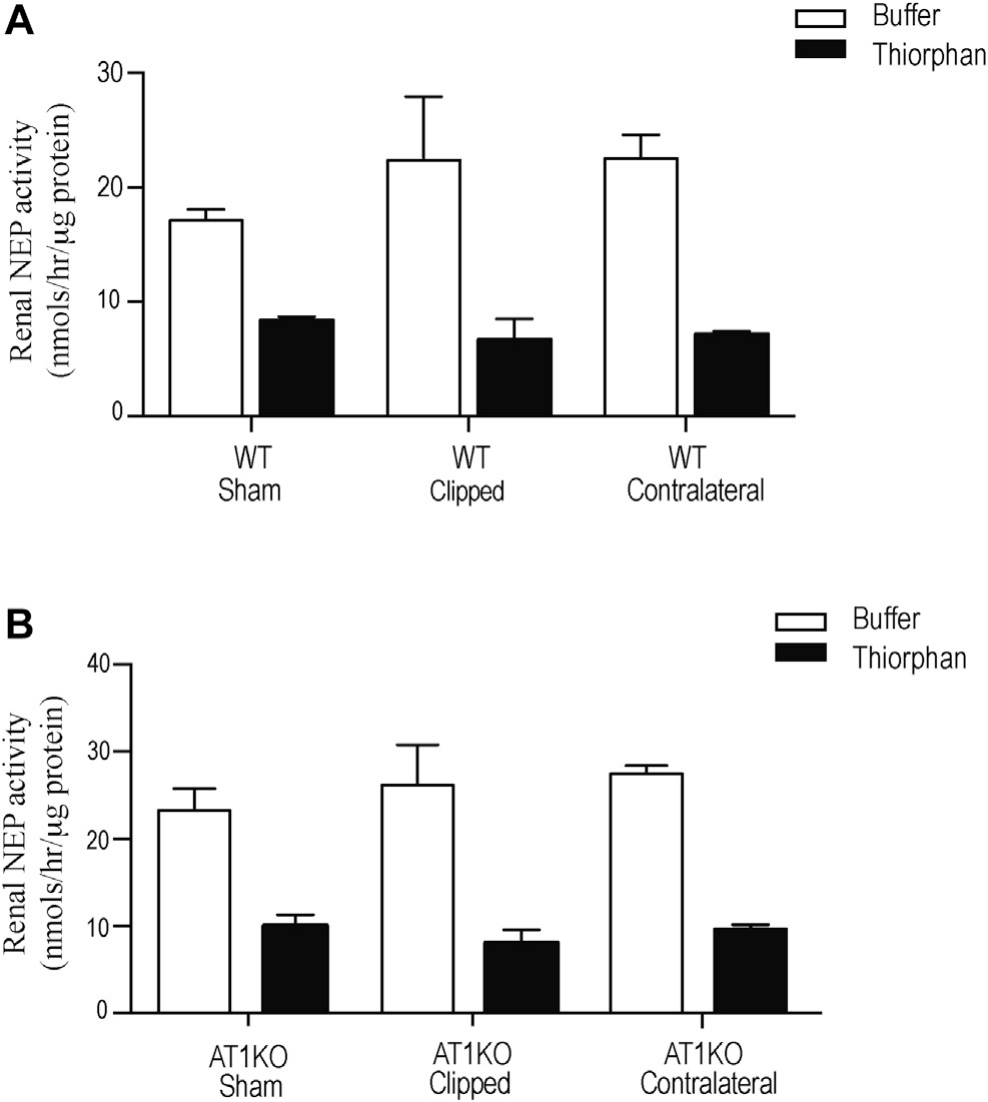
Effect of renovascular hypertension on Renal NEP activity in Sham, Clipped and Contralateral kidneys using fluorogenic enzyme assay: Renal NEP activity was measured using 1 µg of kidney samples and fluorogenic NEP substrate in presence and absence of the NEP inhibitor thiophen. Each bar represents mean ± SEM (n = 6–8).

Renal NEP activity was measured using the fluorogenic substrate, Suc-Ala-Ala-Phe-AMC in the presence and absence of the NEP inhibitor, thiophen as described before (Hosawi et al., 2019). Figure 8 shows no significant difference in renal NEP activity between clipped kidney and sham-operated controls. Furthermore, there was no significant difference in renal NEP activity between the contralateral kidneys and the sham-operated controls (Figure 8).

### Urinary ACE2 and NEP Activities Were Not Altered After 2K1C

**FIGURE 9:**
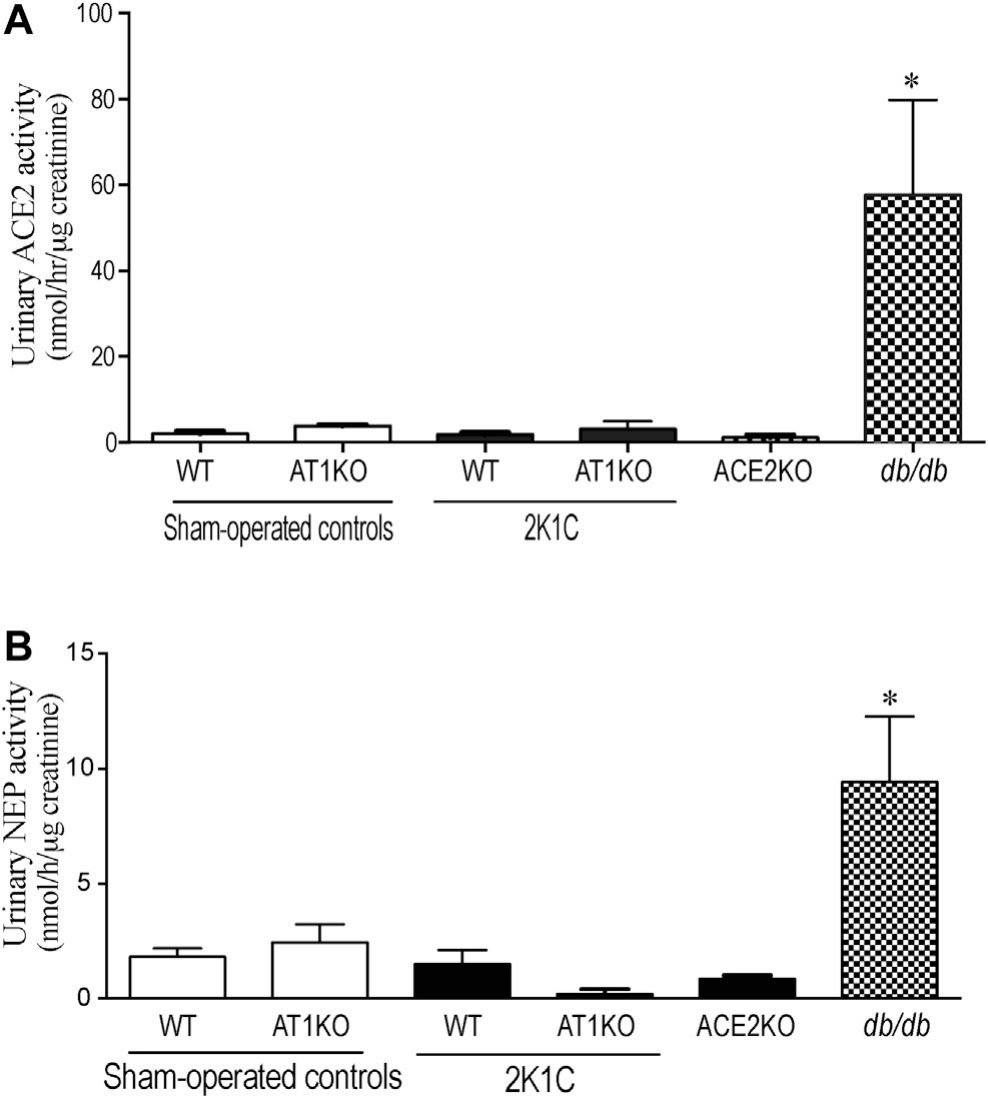
Effect of renovascular hypertension on urinary ACE2 and NEP activity in WT and AT1KO mice after 2 weeks of 2K1C Goldblatt surgery. **(A)** urinary ACE2 activity in WT (sham), WT (2K1C), AT1KO (sham), AT1KO (2K1C) was measured using 6–10 µl urine samples. Urine samples from *db*/*db* diabetic mice were used as positive control. The experiment was validated using urine samples from ACE2KO mice and ACE2 inhibitor MLN-4670. **(B)** urinary NEP activity was measured in WT (sham), WT (2K1C), AT1KO (sham), AT1KO (2K1C) using 6–10 µl urine samples. Urine samples from *db*/*db* diabetic mice were used as positive control. The experiment was validated using kidney samples from NEPKO mice and the NEP inhibitor Thiorphan. *p < 0.0001 vs. buffer. Each bar represents mean ± SEM (n = 6–8).

ACE2 and NEP activity were measured in 24-h urine samples collected from 2K1C and sham-operated mice. For urinary ACE2 activity, Mca-APK (Dnp) was used as substrate in the presence and absence of the ACE2 inhibitor, MLN-460 as describe before (Alawi et al., 2020). In addition, the assay was validated by using urine samples from *db*/*db* mice as a positive control and ACE2KO mice as negative control (Figure 9A). As expected, no detectable ACE2 was observed in the urine of ACE2KO mice. For urinary NEP activity, Suc-Ala-Ala-Phe-AMC was used as substrate in the presence and absence of the NEP inhibitor, thiophen as described before (Hosawi et al., 2019). The urinary ACE2 activity (Figure 9A) and urinary NEP activity (Figure 9B) detected in WT and AK1KO were almost 50-folds less than those observed with *db*/*db* urine samples. 2K1C Goldblatt surgery had no effect on urinary ACE2 and NEP activity in WT and AT1KO mice during the 2 weeks of the study.

### Modulation of Urinary ACE2 and NEP Protein Expression in 2K1C and *db*/*db* Mice:

**FIGURE 10:**
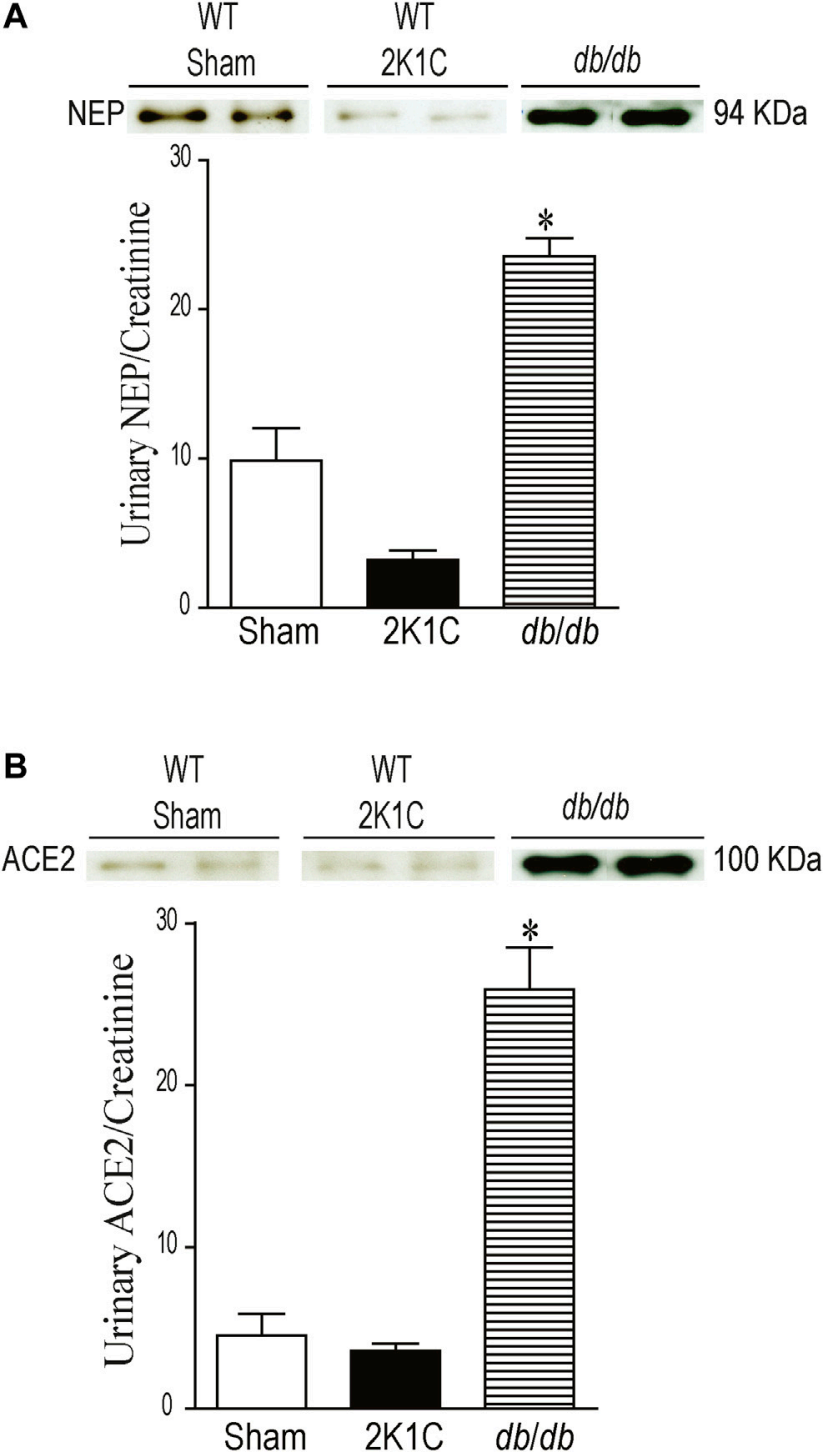
Urine volumes equivalent to 0.75 µg creatinine was used for western blot analysis of urinary ACE2 **(A)** and NEP **(B)** expression in WT mice. Urine samples from *db*/*db* diabetic mice was used as positive controls. In each individual experiment, samples were loaded randomly in four gels. Two Immunoreactive bands from each group corresponding to NEP and ACE2 were appropriately cropped and spliced as a representative of the result. In *db*/*db* mice, ACE2 was detected at 100 kDa, whereas only faint bands for ACE2 were detected in the urine of sham and after 2K1C surgery. In *db*/*db* and WT (Sham) mice, full length NEP was detected at 94 kDa, whereas decreased expression of NEP was detected in the urine of WT mice after 2K1C surgery. *p < 0.001 vs. WT (sham), Each bar represents mean ± SEM (n = 6–8).

Urinary NEP and ACE2 Western blot analyses were performed using urine sample equivalent to 0.75 μg creatinine. Urine samples from *db*/*db* mice were used as a positive control. Immunoreactive band for urinary NEP was clearly detected at a molecular weight ≈95 KDa in WT sham-operated and WT 2K1C mice (Figure 10A, *p* < 0.001). The urinary expression of NEP was significantly decreased in WT 2K1C compared with sham-operated controls (Figure 10A, *p* < 0.001). Urinary NEP in AT1KO 2K1C was also significantly decreased compared with AT1KO sham-operated (data not shown). Unlike urinary NEP, there was only faint immunoreactive bands for ACE2 in WT sham and WT 2K1C (Figure 10B). However, there was an upregulation of urinary ACE2 in *db*/*db* mice compared with WT sham (Figure 10B), which agrees with previous studies (Chodavarapu et al., 2013). There was no significant difference between urinary ACE2 in AT1KO 2K1C and AT1KO sham-operated (data not shown).

### Urinary KIM-1 Protein Expression Was Increased in 2K1C Mice

**FIGURE 11:**
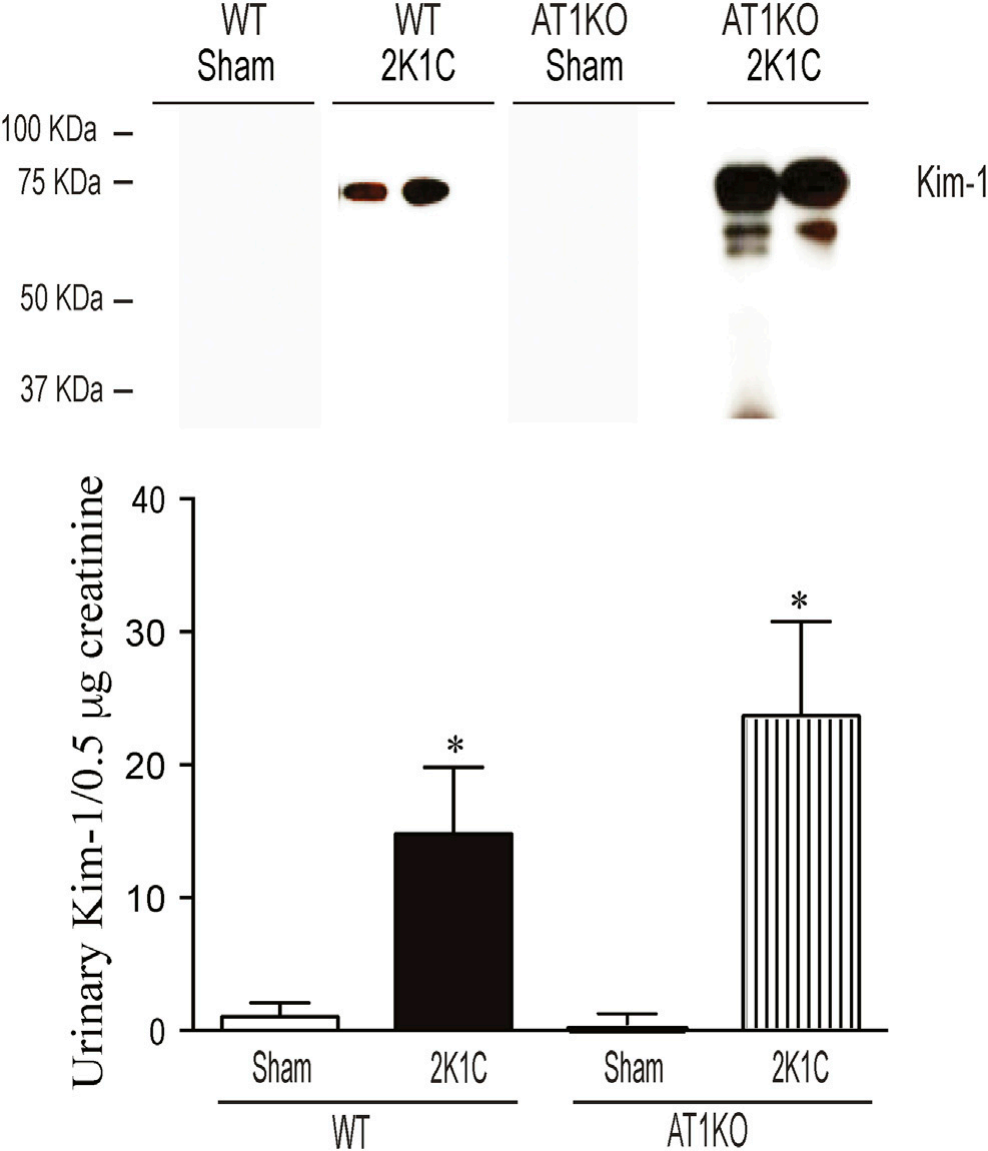
Immunoblotting revealed increased expression of KIM-1 in the urine of WT and AT1KO mice undergoing 2K1C surgery. Urine volumes equivalent to 0. 5 µg creatinine was used for western blot analysis of urinary KIM-1 expression in the urine of WT and AT1KO mice undergoing 2K1C surgery. In each individual experiment, samples were loaded randomly in four gels. Two Immunoreactive bands from each group corresponding to KIM-1 were appropriately cropped and spliced as a representative of the result. In 2K1C mice, KIM-1 was detected at 75 kDa, whereas only faint bands for KIM-1 were detected in the urine of sham. *p < 0.001 vs. Sham Each bar represents mean ± SEM (n = 6–8).

The urinary expression of KIM-1 was significantly increased in WT 2K1C and AT1KO 2K1C compared with sham-operated controls (Figure 11, *p* < 0.001). As expected, there was hardly no evidence for urinary KIM-1 in sham-operated controls. It is interesting that there was a significant increase of urinary KIM-1 in AT1KO 2K1C compared to WT 2K1C (Figure 11, *p* < 0.05).

### Kidney Histology and Immunostaining

To support the enzyme activity and western blot data, we explored immunostaining of kidney sections for ACE2, NEP, KIM-1 and markers for kidney injury. Renal mesangial expansion is one of the histological characteristics of kidney injury. The mesangial matrix was identified by the presence of PAS-positive and nuclei-free areas in the mesangial. Glomerular tufts of clipped and contralateral WT kidneys revealed a significant increase in the mesangial expansion compared to the sham-operated WT controls (Supplementary Figure S1B, *p* < 0.001). In AT1KO mice, the relative % mesangial matrix area was significantly decreased in the clipped kidneys compared with the clipped kidneys of WT mice (Supplementary Figure S1B, *p* < 0.001). Masson's Trichome staining reveals collagen deposition as blue color. Semi quantitative analysis of Masson Trichome staining demonstrated a significant increase of interstitial tubular fibrosis in WT and AT1KO 3 weeks following 2K1C Goldblatt surgery (Supplementary Figure S2). In AT1KO mice, interstitial tubular fibrosis was significantly decreased in the clipped kidneys compared with the clipped kidneys of WT mice (Supplementary Figure S2B, *p* < 0.001).

**FIGURE 12:**
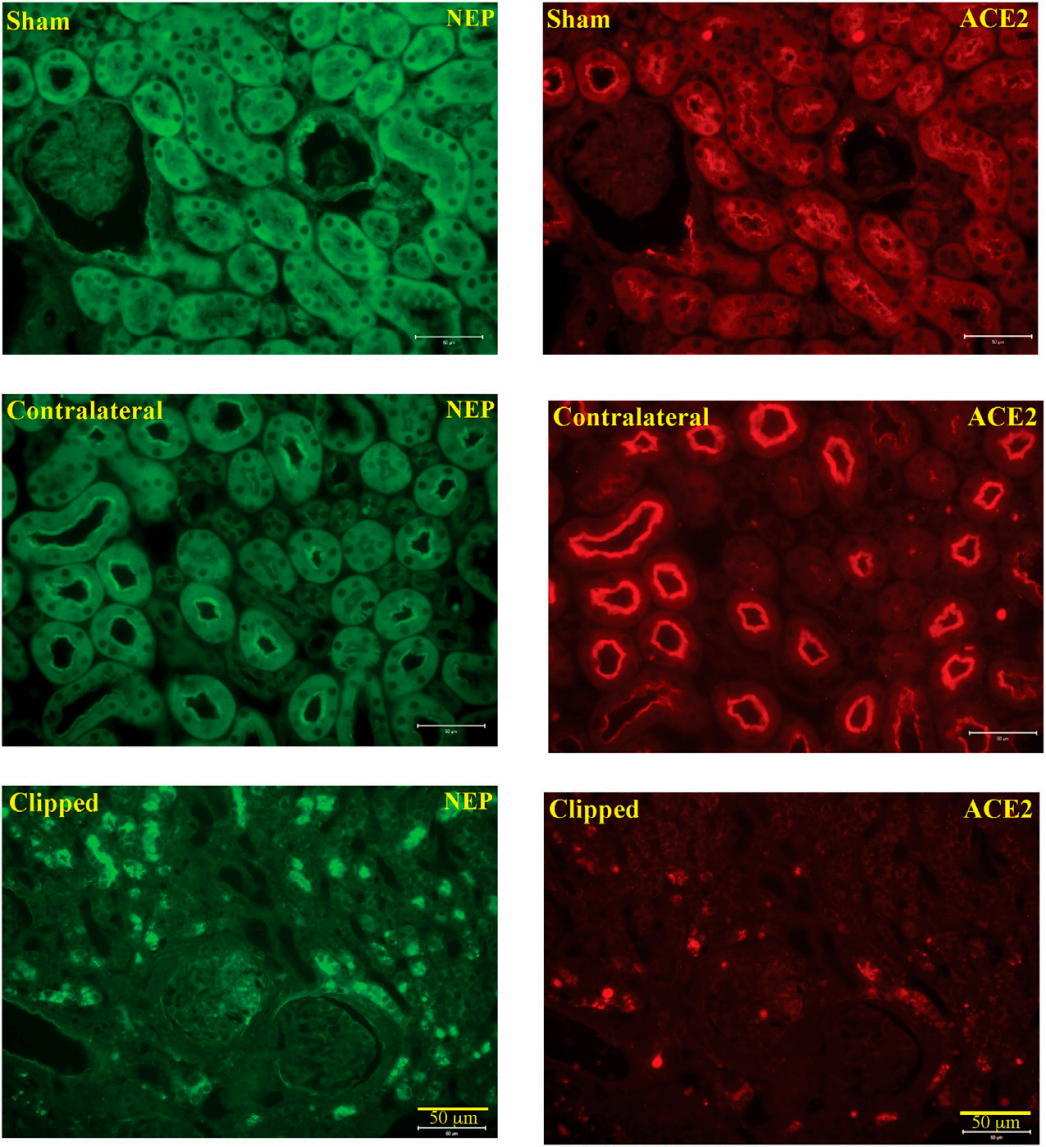
Representative light microscopy images of immunofluorescence staining for NEP (FITC, green fluorescence) and ACE2 (cy3, red fluorescence) in section of sham, clipped and contralateral kidneys obtained from WT mice. Magnification: ×20, scale bars:50 μm.

**FIGURE 13:**
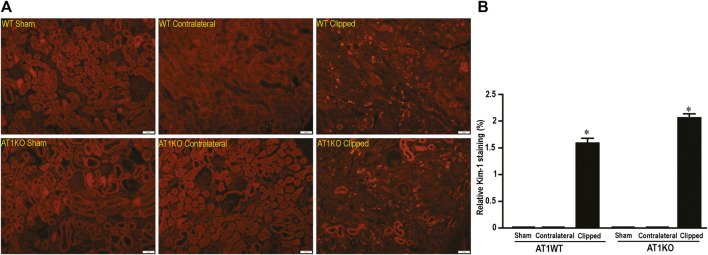
Representative light microscopy images of kidney section from sham, clipped and contralateral kidney from WT and AT1KO mice stained with KIM-1. Magnification: ×20, scale bars: 50 μm. **(B)** Semiquantitative scores of KIM-1 analyzed by Metamorph. There was a significant increase of renal KIM-1 expression in the clipped kidneys of both WT and AT1KO mice. Each bar represents mean ± SEM. (n = 4–5, *p < 0.001 vs. sham controls).± ±

In the kidney, NEP and ACE2 protein expression were observed mainly in the brush border of the renal tubules of the WT mice and to a lesser extend in the glomerulus. Figure 12 shows immunofluorescence of NEP (Figure 12, green fluorescence) and immunofluorescence of ACE2 (Figure 12, red fluorescence) in WT mice (sham, contralateral and clipped kidneys). Figure 13 shows the immunofluorescence staining of renal KIM-1 expression in kidney section. There was no evidence for immunostaining of KIM-1 in sham and contralateral kidney sections in WT and AT1KO mice. There was a significant increase of immunostaining of KIM-1 in clipped kidneys in both WT and AT1KO mice (Figure 13, *p* < 0.001).

## Discussion

The local intrarenal RAS functions as a paracrine hormonal system independent from the systemic RAS, and accumulating evidence support its pivotal role in cardiovascular and renal diseases (Culver et al., 2017). The aim of the study was to evaluate the intrarenal and urinary ACE2 and NEP in the 2K1C Goldblatt model of renovascular hypertension and test the hypothesis that they are elevated and could be utilized as early biomarkers for kidney injury. In addition, we investigated the impact of global deletion of the AT1R on intrarenal ACE2, NEP, and the known marker of acute kidney marker, KIM-1. The present study demonstrates the major new finding that 2K1C Goldblatt renovascular hypertensive model in WT and AT1KO mice is associated with a significant loss of renal ACE2 and NEP protein expression and activity. This loss of renal NEP correlates with a significant decrease of urinary ACE2 and NEP expression and activity in 2K1C mice. The data suggests that urinary NEP mirrors intrarenal NEP status. One of the novel findings is that there was measurable urinary NEP activity and expression in the WT mice while the urinary ACE2 in WT and 2K1C was almost undetectable. Although deleting AT1R decreased albuminuria and hypertension in the 2K1C Goldblatt model, it did not protect against the decreased renal ACE2 and NEP expression and activity. Another novel finding is that urinary KIM-1 expression was increased following the clipping procedures in WT and AT1KO mice. Again, this increased urinary KIM-1 expression was not attenuated by deleting AT1R.

In the present study, immunofluorescence techniques confirmed the predominant expression of ACE2 and NEP in the apical side of the renal proximal tubules, which might be of great relevance to the finding that both ACE2 and NEP are detected in the urine.

Urinary albumin excretion and estimated glomerular filtration rate (eGFR) are considered the gold standard indicators for the diagnosis and assessment of CKD (Currie, 2014). However, there are some cases presented with renal dysfunction but without microalbuminuria, indicating the insensitivity of these biomarkers, especially in the early stages of renal disease (Lee and Choi, 2015). Therefore, there is a need for sensitive urinary biomarkers which could aid in early detection and proper management of the care for patients with acute kidney injury and CKD. There is active research on some of the proposed candidates of urinary biomarkers, and among these molecules are NGAL, angiotensinogen, renin, and NEP (Liu et al., 2013; Yang et al., 2015; Juretzko et al., 2017; Pajenda et al., 2017; Salih et al., 2017; Wysocki et al., 2017).

Recent studies have shown increased urinary ACE2 levels and activity in type 1 (Cherney et al., 2014; Burns et al., 2017) and type 2 diabetic patients (Park et al., 2013b; Liang et al., 2015; Mariana et al., 2016) and diabetic mice (Wysocki et al., 2013). In addition, an increase in the urinary ACE2 expression and activity was demonstrated in patients with diabetic and IgA nephropathies, thus adding value to its diagnostic potential (Mizuiri et al., 2011; Abe et al., 2015). Continuing in this vein, our laboratory has shown increased urinary ACE2 activity and expression in the urine of type 1 (Salem et al., 2014) and type 2 diabetic mice (Chodavarapu et al., 2013; Somineni et al., 2014) and of type 2 diabetic patients (Gutta et al., 2018). The functional significance and mechanism of regulation of intrarenal ACE2 and NEP under an environment of hypertension is still unclear and has not yet been fully elucidated. Previous studies suggest that a decrease in intrarenal ACE2 in the first 2 weeks might play a role in the development of hypertension in 2K1C rats (Kim et al., 2016). In light of the above data in diabetic mice and patients (Mizuiri et al., 2011; Gutta et al., 2018), we hypothesized that kidney injury and hypertension in the 2K1C Goldblatt model could also increase the shedding of ACE2 and NEP into the urine, and deleting AT1R will attenuate this effect. In the present study, we compared urinary ACE2 shedding in *db*/*db* diabetic model of diabetic kidney disease and 2K1C Goldblatt model of renovascular hypertension. We demonstrated that only diabetic kidney disease in *db*/*db* is associated with increased urinary ACE2 shedding. In contrast to diabetic kidney disease in *db*/*db* mice, we have noticed no detectable urinary ACE2 and NEP expression and activity following the clipping procedures in mice. In addition, we showed significant loss of renal ACE2 and NEP protein expression in the clipped kidney in both WT and AT1KO mice compared with the contralateral and sham-operated kidneys. During the early phase of hypertension, the contralateral kidney is exposed to high blood pressure, which causes kidney injury (Polichnowski and Cowley, 2009). However, our data showed no alteration in the renal ACE2 and NEP expression in the contralateral kidney compared to the sham and clipped ones. This might suggest that the modulation of NEP and ACE2 is independent of renovascular hypertension after 2 weeks of 2K1C Goldblatt surgery, since it occurred in both the WT hypertensive and normotensive AT1KO mice.

One objective of the present study was to investigate and reconfirm the relative contribution of AT1R in the development of hypertension in the 2K1C Goldblatt mouse model and its impact on renal and urinary ACE2 and NEP protein expression. Several studies have focused on the most active peptide of the intrarenal RAS, Ang II, due to its critical role in hypertension and renal injury. The elevation of blood pressure is mainly mediated by the vasoconstrictor Ang II when it binds to its receptor, AT1R (De Gasparo et al., 2000). Two subtypes of AT1R have been cloned in mice and rats, AT1a and AT1b, and AT1aR has been considered the dominant subtype in most organs (Sasamura et al., 1992). We used an AT1aR null mouse model (AT1KO) to determine its relative contribution to hypertension and shedding of RAS candidates. In the study, there was a significant decrease in blood pressure in AT1KO mice at baseline, which is in agreement with previous studies (Oliverio et al., 2000; Cervenka et al., 2002; Gurley et al., 2011). As expected, WT mice showed a robust increase in MAP after placement of the unilateral renal clip compared with sham-operated mice throughout the 2 weeks of the study. In contrast, we observed no change in MAP of AT1KO mice after 2K1C surgery. The blood pressure remained normotensive in AT1KO after 2K1C throughout the duration of the 2 weeks. These data provide strong evidence in support of the notion that the AT1R plays a role in the pathogenesis of renovascular hypertension, which corresponds with a study that reported the predominate role of AT1R in the 2K1C model (Cervenka et al., 2008). Whaley-Connell *et al.* showed that treatment of the hypertensive transgenic Ren2 rats with the AT1R blocker valsartan increases renal ACE2 and NEP expression, suggesting a contribution of renal RAS components to the renoprotective effects of valsartan (Whaley-Connell et al., 2006). However, our data demonstrated that high blood pressure and albuminuria are independent of ACE2 shedding, since we found that AT1R had no effect on the expression of either renal or urinary ACE2 protein expression and activity. The down-regulation of urinary ACE2 protein excretion that we observed in 2K1C mice may reflect the renal ACE2 status. These findings of decreased renal and urinary ACE2 in this model of hypertension do not support our original hypothesis and previous findings of increased shedding of ACE2 in mouse model of diabetic nephropathy (Chodavarapu et al., 2013; Salem et al., 2014; Somineni et al., 2014). However, they are in agreement with a study on 2K1C rats which employed western blot and immunohistochemistry to demonstrate that ACE2 is attenuated in the early stages of hypertension (Kim et al., 2016), and a study on subtotal nephrectomized (STNx) rats, which showed that renal ACE2 activity is reduced in acute kidney injury, which contributes to the development of CKD (Velskoska et al., 2010). We could conclude from these results that the presence of high levels of urinary ACE2 in the *db*/*db* diabetic models and low levels in 2K1C Goldblatt and STNx models may indicate the specificity of ACE2 as a biomarker mainly for the prognosis of renal injury in diabetes.

The high blood pressure in the WT mice was followed by increased albuminuria from the first week of the unilateral renal clipping. This increase in urinary albumin excretion was significant after one week when compared with the baseline and sham-operated groups. At the end of the two-week study period, the 2K1C mice excreted more urinary albumin than the first week of the surgery, which indicates that albuminuria increased with the progression of renal injury and advanced age. However, deleting the AT1R attenuated albuminuria in these mice showed the same trend as seen in blood pressure. This data determines the pivotal role of AT1 receptor in the development of albuminuria in this hypertensive model.

NEP has been reported in several studies to be involved in the degradation of various active peptides such as Ang I, bradykinin, natriuretic peptides (Judge et al., 2015), and beta amyloid (Park et al., 2013a). Previous studies have shown an increase in the shedding of urinary NEP in diabetic patients with microalbuminuria (Gutta et al., 2018). In this study, we also investigated whether NEP is modulated by kidney injury in the 2K1C Goldblatt model. The data demonstrated a significant decrease of renal NEP protein expression in the clipped kidney of the WT and AT1KO group compared with the contralateral and sham-operated kidneys. Since 2K1C is a well-known high renin model, and our data confirmed the increased renin protein expression in the contralateral kidneys, we anticipated less production of Ang-(1–7) *via* NEP, which could lead to kidney injury. However, in the contralateral kidney of 2K1C, there was no alteration of NEP content in both mice WT and AT1KO compared to the WT sham controls, and this could be a feedback mechanism from high levels of Ang one and Ang II.

As expected, the expression of renin was increased in the clipped kidney of wild type mice compared with the contralateral kidney and the sham control. The results were in agreement with a previous study where the increased renin activity during first weeks of renal placement was normalized after 11 weeks post-surgery (Grobe et al., 2015). The studies in hypertensive rats also showed an increase in the expression of renin in the clipped kidney after 2 weeks of clip placement (Kim et al., 2016). In the 2K1C model, the decrease in the renal perfusion pressure caused by unilateral clipping of renal artery led to an increase in renin synthesis, which in turn increased the circulation of Ang II (Kobori et al., 2007).

In contrast to diabetes, there was no detectable urinary ACE2 and NEP expression and activity in 2K1C mice. This could be due the fact the level of urinary ACE2 and NEP are already low/undetectable in sham-operated controls compared to *db*/*db* diabetic mice. However, urinary KIM-1 expression was significantly increased in the 2K1C mice in both the WT and AT1KO groups, which agrees with the 2K1C-mediated upregulation of the intrarenal KIM-1 protein expression in both WT and AT1KO mice. It is worth noting that in the Masson Trichome staining experiment, there was a significant decrease of tubulo-interstitial fibrosis in the clipped AT1KO kidney compared to the clipped WT kidney. However, the same experiment demonstrated a significant increase of tubulo-interstitial fibrosis in the AT1KO clipped kidney compared to the AT1KO sham-operated control. As expected, deleting AT1R attenuated the development of hypertension and albuminuria in the 2K1C mice. This novel finding suggests that the elevated urinary KIM-1 observed in the AT1KO mice is indicative of kidney injury and is independent of hypertension and albuminuria. This data agrees with a recent clinical study in patients with renal disease, where there was no association between urinary KIM-1 and proteinuria (van Timmeren et al., 2007).

In conclusion, our data showed a loss of renal ACE2 and NEP in 2K1C mice with albuminuria and its association with reduced urinary ACE2 and NEP, which may imply the severity of disease progression at early stages. Our study also provides a unique approach for the investigation of urinary KIM-1 as a sensitive biomarker for CKD. Western blot analysis of urinary RAS suggests a potential mechanistic basis for the degradation of ACE2 and NEP and shedding of their proteolytic fragments into the urine. The finding that AT1R has no effect on renal ACE2 and NEP is a major asset of our study. Taken together, our findings suggest that urinary ACE2 and NEP are not increased during the course of renovascular hypertension in CKD and KIM-1 could be used as a biomarker in this model.

## Data Availability

The raw data supporting the conclusions of this article will be made available by the authors, without undue reservation.

## References

[B1] Abe M. Oikawa O. Okada K. Soma M. (2015). Urinary angiotensin-converting enzyme 2 increases in diabetic nephropathy by angiotensin II type 1 receptor blocker olmesartan. J. Renin-Angiotensin-Aldosterone Syst. 16, 159–164. 10.1177/1470320314551443 25287898

[B2] Alawi L. F. (2015). Role of angiotensin II type 1 A receptors on renal and urinary angiotensin converting enzyme 2 (ACE2) and neprilysin (NEP) in the two-kidney one- clip (2K1C) model of renovascular hypertension. 2 Available at: http://rave.ohiolink.edu/etdc/view?acc_num=wright1432837235 (Accessed November 18, 2020). 10.3389/fphar.2020.602985 PMC7941277 33708117

[B3] Alawi L. F. Emberesh S. E. Owuor B. A. Chodavarapu H. Fadnavis R. El-Amouri S. S. (2020). Effect of hyperglycemia and rosiglitazone on renal and urinary neprilysin in db/db diabetic mice. Physiol. Rep. 8, e14364–13. 10.14814/phy2.14364 32026607 PMC7002536

[B4] Burns K. D. Lytvyn Y. Mahmud F. H. Daneman D. Deda L. Dunger D. B. (2017). The relationship between urinary renin-angiotensin system markers, renal function, and blood pressure in adolescents with type 1 diabetes. Am. J. Physiol. Renal Physiol. 312, F335–F342. 10.1152/ajprenal.00438.2016 27733369

[B5] Cervenka L. Horácek V. Vanecková I. Hubácek J. A. Oliverio M. I. Coffman T. M. (2002). Essential role of AT1A receptor in the development of 2K1C hypertension. Hypertension 40, 735–741. 10.1161/01.hyp.0000036452.28493.74 12411470

[B6] Cervenka L. Vanecková I. Husková Z. Vanourková Z. Erbanová M. Thumová M. (2008). Pivotal role of angiotensin II receptor subtype 1A in the development of two-kidney, one-clip hypertension: study in angiotensin II receptor subtype 1A knockout mice. J. Hypertens. 26, 1379–1389. 10.1097/HJH.0b013e3282fe6eaa 18551014 PMC2704388

[B7] Cherney D. Z. Xiao F. Zimpelmann J. Har R. L. Lai V. Scholey J. W. (2014). Urinary ACE2 in healthy adults and patients with uncomplicated type 1 diabetes. Can. J. Physiol. Pharmacol. 92, 703–706. 10.1139/cjpp-2014-0065 24920267

[B8] Chodavarapu H. Grobe N. Somineni H. K. Salem E. S. Madhu M. Elased K. M. (2013). Rosiglitazone treatment of type 2 diabetic db/db mice attenuates urinary albumin and angiotensin converting enzyme 2 excretion. PloS One 8, e62833. 10.1371/journal.pone.0062833 23646149 PMC3639987

[B9] Culver S. Li C. Siragy H. M. (2017). Intrarenal angiotensin-converting enzyme: the old and the new. Curr. Hypertens. Rep. 19, 80. 10.1007/s11906-017-0778-2 28929450 PMC5913745

[B10] Currie G. McKay G. Delles C. (2014). Biomarkers in diabetic nephropathy: present and future. World J. Diabetes 5, 763. 10.4239/wjd.v5.i6.763 25512779 PMC4265863

[B11] De Gasparo M. Catt K. J. Inagami T. Wright J. W. Unger T. (2000). International union of pharmacology. XXIII. The angiotensin II receptors. Pharmacol. Rev. 52, 415–472. 10977869

[B12] Domenig O. Manzel A. Grobe N. Königshausen E. Kaltenecker C. C. Kovarik J. J. (2016). Neprilysin is a mediator of alternative renin-angiotensin-system Activation in the murine and human kidney. Sci. Rep. 6, 33678. 10.1038/srep33678 27649628 PMC5030486

[B13] Elased K. M. Gutta S. Alawi L. F. Somineni H. K. Boivin G. P. (2013). Effect of exercise and rosiglitazone on neprilysin protein expression in db/db diabetic mice. Diabetologia 56, S308.

[B14] Fang F. Liu G. C. Zhou X. Yang S. Reich H. N. Williams V. (2013). Loss of ACE2 exacerbates murine renal ischemia-reperfusion injury. PloS One 8, e71433. 10.1371/journal.pone.0071433 23951161 PMC3739768

[B15] Furuhashi M. Moniwa N. Mita T. Fuseya T. Ishimura S. Ohno K. (2015). Urinary angiotensin-converting enzyme 2 in hypertensive patients may be increased by olmesartan, an angiotensin II receptor blocker. Am. J. Hypertens. 28, 15–21. 10.1093/ajh/hpu086 24842388

[B16] Goldblatt H. Lynch J. Hanzal R. F. Summerville W. W. (1934). Studies on experimental hypertension : I. The production of persistent elevation of systolic blood pressure by means of renal ischemia. J. Exp. Med. 59, 347–379. 10.1084/jem.59.3.347 19870251 PMC2132360

[B17] Grobe N. Leiva O. Morris M. Elased K. M. (2015). Loss of prolyl carboxypeptidase in two-kidney, one-clip goldblatt hypertensive mice. PloS One 10, e0117899. 10.1371/journal.pone.0117899 25706121 PMC4338234

[B18] Gurley S. B. Riquier-Brison A. D. M. Schnermann J. Sparks M. A. Allen A. M. Haase V. H. (2011). AT1A angiotensin receptors in the renal proximal tubule regulate blood pressure. Cell Metab. 13, 469–475. 10.1016/j.cmet.2011.03.001 21459331 PMC3070917

[B19] Gutta S. Grobe N. Kumbaji M. Osman H. Saklayen M. Li G. (2018). Increased urinary angiotensin converting enzyme 2 and neprilysin in patients with type 2 diabetes. Am. J. Physiol. Renal Physiol. 315, F263–F274. 10.1152/ajprenal.00565.2017 29561187 PMC6139527

[B20] Han W. K. Bailly V. Abichandani R. Thadhani R. Bonventre J. V. (2002). Kidney Injury Molecule-1 (KIM-1): a novel biomarker for human renal proximal tubule injury. Kidney Int. 62, 237–244. 10.1046/j.1523-1755.2002.00433.x 12081583

[B21] Hosawi A. Dhakal S. Thanekar U. Alawi L. Sawant H. Grobe N. (2019). Abstract P2036: effects of angiotensin II type 1 A receptor (AT1aR) on renal and urinary biomarkers of acute kidney injury in two kidney one clip (2K1C) model of renovascular hypertension. Hypertension 74. 10.1161/hyp.74.suppl_1.P2036 PMC7941277 33708117

[B22] Ichimura T. Bonventre J. V. Bailly V. Wei H. Hession C. A. Cate R. L. (1998). Kidney injury molecule-1 (KIM-1), a putative epithelial cell adhesion molecule containing a novel immunoglobulin domain, is up-regulated in renal cells after injury. J. Biol. Chem. 273, 4135–4142. 10.1074/jbc.273.7.4135 9461608

[B23] Judge P. Haynes R. Landray M. J. Baigent C. (2015). Neprilysin inhibition in chronic kidney disease. Nephrol. Dial. Transplant. 30, 738–743. 10.1093/ndt/gfu269 25140014 PMC4425478

[B24] Juretzko A. Steinbach A. Hannemann A. Endlich K. Endlich N. Friedrich N. (2017). Urinary angiotensinogen and renin excretion are associated with chronic kidney disease. Kidney Blood Press. Res. 42, 145–155. 10.1159/000474932 28395289

[B25] Karoor V. Oka M. Walchak S. J. Hersh L. B. Miller Y. E. Dempsey E. C. (2013). Neprilysin regulates pulmonary artery smooth muscle cell phenotype through a platelet-derived growth factor receptor-dependent mechanism. Hypertension 61, 921–930. 10.1161/HYPERTENSIONAHA.111.199588 23381789 PMC3667616

[B26] Kim Y. G. Lee S. H. Kim S. Y. Lee A. Moon J. Y. Jeong K. H. (2016). Sequential activation of the intrarenal renin-angiotensin system in the progression of hypertensive nephropathy in Goldblatt rats. Am. J. Physiol. Renal Physiol. 311, F195–F206. 10.1152/ajprenal.00001.2015 26823279

[B27] Klag M. J. Whelton P. K. Randall B. L. Neaton J. D. Brancati F. L. Ford C. E. (1996). Blood pressure and end-stage renal disease in men. N. Engl. J. Med. 334, 13–18. 10.1056/NEJM199601043340103 7494564

[B28] Kobori H. Nangaku M. Navar L. G. Nishiyama A. (2007). The intrarenal renin-angiotensin system: from physiology to the pathobiology of hypertension and kidney disease. Pharmacol. Rev. 59, 251–287. 10.1124/pr.59.3.3 17878513

[B29] Kuhn J. H. Li W. Choe H. Farzan M. (2004). Angiotensin-converting enzyme 2: a functional receptor for SARS coronavirus. Cell. Mol. Life Sci. 61, 2738–2743. 10.1007/s00018-004-4242-5 15549175 PMC7079798

[B30] Lee S. Y. Choi M. E. (2015). Urinary biomarkers for early diabetic nephropathy: beyond albuminuria. Pediatr. Nephrol. 30, 1063–1075. 10.1007/s00467-014-2888-2 25060761 PMC4305495

[B31] Liang Y. Deng H. Bi S. Cui Z. A L. Zheng D. (2015). Urinary angiotensin converting enzyme 2 increases in patients with type 2 diabetic mellitus. Kidney Blood Press. Res. 40, 101–110. 10.1159/000368486 25791940

[B32] Liu K. D. Yang W. Anderson A. H. Feldman H. I. Demirjian S. Hamano T. (2013). Urine neutrophil gelatinase-associated lipocalin levels do not improve risk prediction of progressive chronic kidney disease. Kidney Int. 83, 909–914. 10.1038/ki.2012.458 23344473 PMC3642209

[B33] Mariana C. P. Ramona P. A. Ioana B. C. Diana M. Claudia R. C. Stefan V. D. (2016). Urinary angiotensin converting enzyme 2 is strongly related to urinary nephrin in type 2 diabetes patients. Int. Urol. Nephrol. 48, 1491–1497. 10.1007/s11255-016-1334-8 27312782

[B34] McMurray J. J.V. Packer M. Desai A. S. Gong J. Lefkowitz M. P. Rizkala A. R. (2014). Angiotensin-neprilysin inhibition versus enalapril in heart failure. N. Engl. J. Med. 371, 993–1004. 10.1056/NEJMoa1409077 25176015

[B35] Mizuiri S. Hemmi H. Arita M. Aoki T. Ohashi Y. Miyagi M. (2011). Increased ACE and decreased ACE2 expression in kidneys from patients with IgA nephropathy. Nephron Clin. Pract. 117, c57–66. 10.1159/000319648 20689326

[B36] Murphy D. McCulloch C. E. Lin F. Banerjee T. Bragg-Gresham J. L. Eberhardt M. S. (2016). Trends in prevalence of chronic kidney disease in the United States. Ann. Intern. Med. 165, 473–481. 10.7326/M16-0273 27479614 PMC5552458

[B37] Núñez J. Núñez E. Miñana G. Carratalá A. Sanchis J. Lupón J. (2016). Serum neprilysin and recurrent hospitalizations after acute heart failure. Int. J. Cardiol. 220, 742–744. 10.1016/j.ijcard.2016.06.271 27393859

[B38] Oliverio M. I. Best C. F. Smithies O. Coffman T. M. (2000). Regulation of sodium balance and blood pressure by the AT(1A) receptor for angiotensin II. Hypertension 35, 550–554. 10.1161/01.HYP.35.2.550 10679496

[B39] Oudit G. Y. Herzenberg A. M. Kassiri Z. Wong D. Reich H. Khokha R. (2006). Loss of angiotensin-converting enzyme-2 leads to the late development of angiotensin II-dependent glomerulosclerosis. Am. J. Pathol. 168, 1808–1820. 10.2353/ajpath.2006.051091 16723697 PMC1606622

[B40] Pajenda S. Mechtler K. Wagner L. (2017). Urinary neprilysin in the critically ill patient. BMC Nephrol. 18, 172. 10.1186/s12882-017-0587-5 28545475 PMC5445475

[B41] Park M. H. Lee J. K. Choi S. Ahn J. Jin H. K. Park J. S. (2013a). Recombinant soluble neprilysin reduces amyloid-beta accumulation and improves memory impairment in Alzheimer’s disease mice. Brain Res. 1529, 113–124. 10.1016/j.brainres.2013.05.045 23831521

[B42] Park S. E. Kim W. J. Park S. W. Park J. W. Lee N. Park C. Y. (2013b). High urinary ACE2 concentrations are associated with severity of glucose intolerance and microalbuminuria. Eur. J. Endocrinol. 168, 203–210. 10.1530/EJE-12-0782 23144053

[B43] Pavo N. Arfsten H. Cho A. Goliasch G. Bartko P. E. Wurm R. (2019). The circulating form of neprilysin is not a general biomarker for overall survival in treatment-naïve cancer patients. Sci. Rep. 9, 2554. 10.1038/s41598-019-38867-2 30796257 PMC6385211

[B44] Polichnowski A. J. Cowley A. W. (2009). Pressure-induced renal injury in angiotensin II versus norepinephrine-induced hypertensive rats. Hypertension 54, 1269–1277. 10.1161/HYPERTENSIONAHA.109.139287 19858406 PMC2812436

[B45] Prieto M. C. González-Villalobos R. A. Botros F. T. Martin V. L. Pagán J. Satou R. (2011). Reciprocal changes in renal ACE/ANG II and ACE2/ANG 1-7 are associated with enhanced collecting duct renin in Goldblatt hypertensive rats. Am. J. Physiol. Ren. Physiol. 300, F749–F755. 10.1152/ajprenal.00383.2009 PMC3064128 21209009

[B46] Prieto-Carrasquero M. C. Botros F. T. Pagan J. Kobori H. Seth D. M. Casarini D. E. (2008). Collecting duct renin is upregulated in both kidneys of 2-kidney, 1-clip Goldblatt hypertensive rats. Hypertension 51, 1590–1596. 10.1161/HYPERTENSIONAHA.108.110916 18426992 PMC2601698

[B47] Sakakibara T. Ura N. Shimamoto K. Ogata H. Ando T. Fukuyama S. (1989). Localization of neutral endopeptidase in the kidney determined by the stop-flow method. Adv. Exp. Med. Biol. 247B, 349–353. 10.1007/978-1-4615-9546-5_58 2558509

[B48] Saito E. S. Grobe N. Elased K. M. (2014). Insulin treatment attenuates renal ADAM17 and ACE2 shedding in diabetic Akita mice. Am. J. Physiol. Renal Physiol. 306, F629–F639. 10.1152/ajprenal.00516.2013 24452639 PMC3949038

[B49] Salih M. Bovée D. M. Roksnoer L. C. W. Casteleijn N. F. Bakker S. J. L. Gansevoort R. T. (2017). Urinary renin-angiotensin markers in polycystic kidney disease. Am. J. Physiol. Renal Physiol. 313, F874–F881. 10.1152/ajprenal.00209.2017 28747358

[B50] Sasamura H. Hein L. Krieger J. E. Pratt R. E. Kobilka B. K. Dzau V. J. (1992). Cloning, characterization, and expression of two angiotensin receptor (AT-1) isoforms from the mouse genome. Biochem. Biophys. Res. Commun. 185, 253–259. 10.1016/S0006-291X(05)80983-0 1599461

[B51] Soler M. J. Wysocki J. Ye M. Lloveras J. Kanwar Y. Batlle D. (2007). ACE2 inhibition worsens glomerular injury in association with increased ACE expression in streptozotocin-induced diabetic mice. Kidney Int. 72, 614–623. 10.1038/sj.ki.5002373 17579661

[B52] Somineni H. K. Boivin G. P. Elased K. M. (2014). Daily exercise training protects against albuminuria and angiotensin converting enzyme 2 shedding in db/db diabetic mice. J. Endocrinol. 221, 235–251. 10.1530/JOE-13-0532 24756098 PMC4004628

[B53] Tipnis S. R. Hooper N. M. Hyde R. Karran E. Christie G. Turner A. J. (2000). A human homolog of angiotensin-converting enzyme. Cloning and functional expression as a captopril-insensitive carboxypeptidase. J. Biol. Chem. 275, 33238–33243. 10.1074/jbc.M002615200 10924499

[B54] van Timmeren M. van den Heuvel M. Bailly V. Bakker S. van Goor H. Stegeman C. (2007). Tubular kidney injury molecule-1 (KIM-1) in human renal disease. J. Pathol. 212, 209–217. 10.1002/path.2175 17471468

[B55] Velkoska E. Dean R. G. Burchill L. Levidiotis V. Burrell L. M. (2010). Reduction in renal ACE2 expression in subtotal nephrectomy in rats is ameliorated with ACE inhibition. Clin. Sci. 118, 269–279. 10.1042/CS20090318 PMC2782317 19698082

[B56] Voors A. A. Gori M. Liu L. C.Y. Claggett B. Zile M. R. Pieske B. (2015). Renal effects of the angiotensin receptor neprilysin inhibitor LCZ696 in patients with heart failure and preserved ejection fraction. Eur. J. Heart Fail. 17, 510–517. 10.1002/ejhf.232 25657064

[B57] Whaley-Connell A. T. Chowdhury N. A. Hayden M. R. Stump C. S. Habibi J. Wiedmeyer C. E. (2006). Oxidative stress and glomerular filtration barrier injury: role of the renin-angiotensin system in the Ren2 transgenic rat. Am. J. Physiol. Renal Physiol. 291, F1308–F1314. 10.1152/ajprenal.00167.2006 16788142

[B58] Wilcox C. S. Cardozo J. Welch W. J. (1996). AT1 and TxA2/PGH2 receptors maintain hypertension throughout 2K,1C Goldblatt hypertension in the rat. Am. J. Physiol. 271, R891–R896. 10.1152/ajpregu.1996.271.4.R891 8897978

[B59] Wong D. W. Oudit G. Y. Reich H. Kassiri Z. Zhou J. Liu Q. C. (2007). Loss of Angiotensin-converting enzyme-2 (Ace2) accelerates diabetic kidney injury. Am. J. Pathol. 171, 438–451. 10.2353/ajpath.2007.060977 17600118 PMC1934545

[B60] Wysocki J. Garcia-Halpin L. Ye M. Maier C. Sowers K. Burns K. D. (2013). Regulation of urinary ACE2 in diabetic mice. Am. J. Physiol. Renal Physiol. 305, F600–F611. 10.1152/ajprenal.00600.2012 23761674 PMC3891267

[B61] Wysocki J. Goodling A. Burgaya M. Whitlock K. Ruzinski J. Batlle D. (2017). Urine RAS components in mice and people with type 1 diabetes and chronic kidney disease. Am. J. Physiol. Renal Physiol., 313, F487–F494. 10.1152/ajprenal.00074.2017 28468961 PMC5582909

[B62] Yamamoto K. Chappell M. C. Brosnihan K. B. Ferrario C. M. (1992). *In vivo* metabolism of angiotensin I by neutral endopeptidase (EC 3.4.24.11) in spontaneously hypertensive rats, Hypertension 19, 692–696. 10.1161/01.hyp.19.6.692 1317352

[B63] Yang X. Chen C. Tian J. Zha Y. Xiong Y. Sun Z. (2015). Urinary angiotensinogen level predicts aki in acute decompensated heart failure: a prospective, two-stage study. J. Am. Soc. Nephrol. 26, 2032–2041. 10.1681/ASN.2014040408 25722365 PMC4520164

[B64] Ye M. Wysocki J. William J. Soler M. J. Cokic I. Batlle D. (2006). Glomerular localization and expression of Angiotensin-converting enzyme 2 and Angiotensin-converting enzyme: implications for albuminuria in diabetes. J. Am. Soc. Nephrol. 17, 3067–3075. 10.1681/ASN.2006050423 17021266

[B65] Zhou P. Yang X. L. Wang X. G. Hu B. Zhang L. Zhang W. (2020). A pneumonia outbreak associated with a new coronavirus of probable bat origin. Nature 579, 270–273. 10.1038/s41586-020-2012-7 32015507 PMC7095418

