# Supplementary material for: Effects of Angiotensin II Type 1A Receptor on ACE2, Neprilysin and KIM-1 in Two Kidney One Clip (2K1C) Model of Renovascular Hypertension
Source: Front Pharmacol. 2021 Jan 29;11:602985. doi: 10.3389/fphar.2020.602985 (PMC7941277; doi:10.3389/fphar.2020.602985)
Supplement: Supplementary file 1 [file datasheet1.pdf]

Supplementary Figure Legends:

Supplementary Figure S1: (A) Representative light microscopy images of kidney section from sham, contralateral, and clipped kidney of WT and AT1KO mice stained with periodic acid-Schiff (PAS). Yellow arrows in the PAS staining show prominent mesangial matrix expansion. Magnification: 40X, scale bars:100 µm. (B) Relative mesangial matrix area (%) significantly increased in clipped and contralateral sections in both WT and AT1KO mice. Each bar represents mean ± SEM (n= 6, $^{*}P<0.01$ & $^{**}P<0.001$ *vs* WT shams; $^{\#}P<0.001$ *vs* AT1KO sham, $^{\$}P<0.001$ vs WT clipped).

Supplementary Figure S2: (A) Representative light microscopy images of kidney section from WT sham, clipped and contralateral kidney from WT and AT1KO mice stained with Masson's Trichrome staining. Masson's Trichome staining reveals collagen deposition (blue color).The Magnification: 20X, scale bars:100 µm. (*B)* Semiquantitative scores of interstitial renal fibrosis index is analyzed by Metamorph. Yellow arrows show more collagen deposit in the renal interstitial of the clipped and contralateral kidney compared to Sham Controls. Each bar represents mean ± SEM. (n=4-5, $*P<0.01$ and $**P<0.001$ *vs* sham WT; $^{\#}P<0.001$ *vs* AT1KO sham, $^{\$}P<0.001$ vs WT clipped).
