# Supplementary figures and images for: Effects of Angiotensin II Type 1A Receptor on ACE2, Neprilysin and KIM-1 in Two Kidney One Clip (2K1C) Model of Renovascular Hypertension
Source: Front Pharmacol. 2021 Jan 29;11:602985. doi: 10.3389/fphar.2020.602985 (PMC7941277; doi:10.3389/fphar.2020.602985)

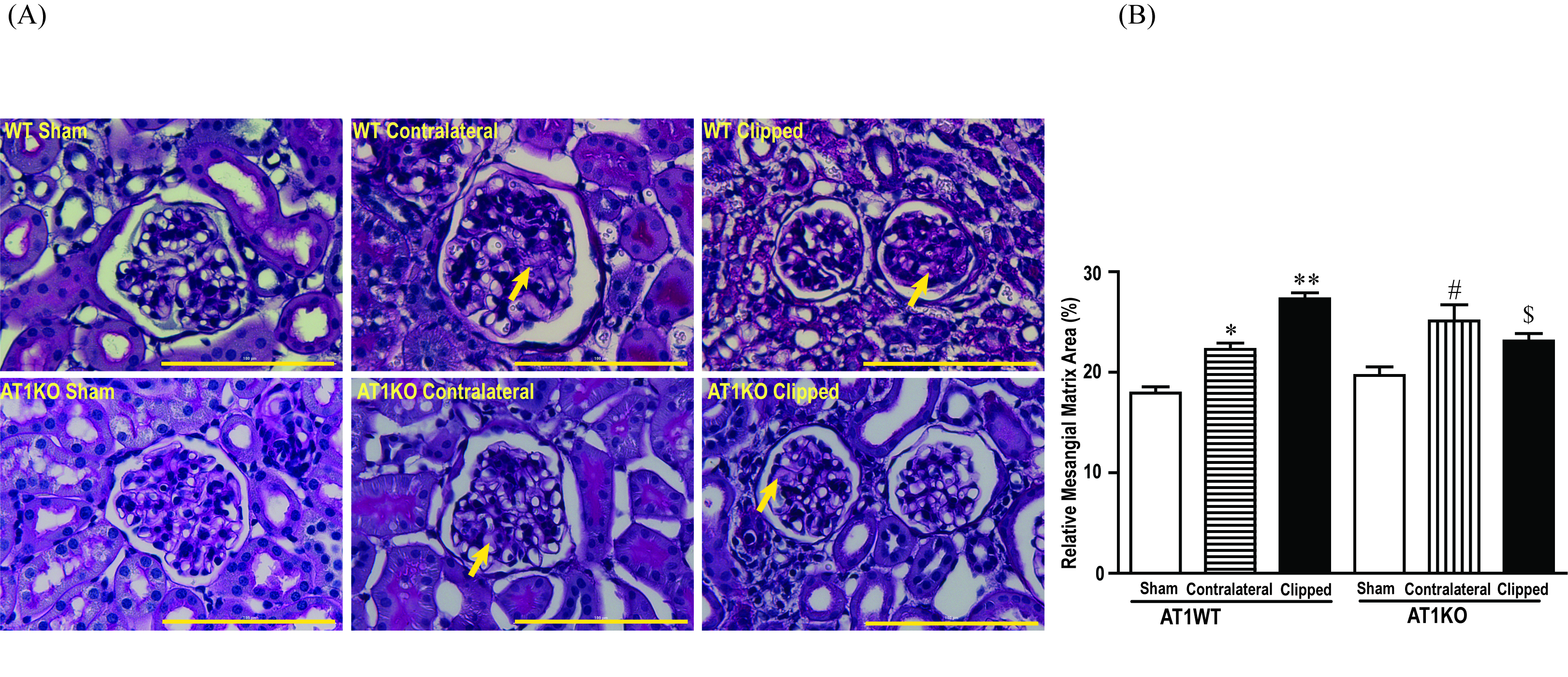

Supplement: Supplementary file 2 [file image1.tif]

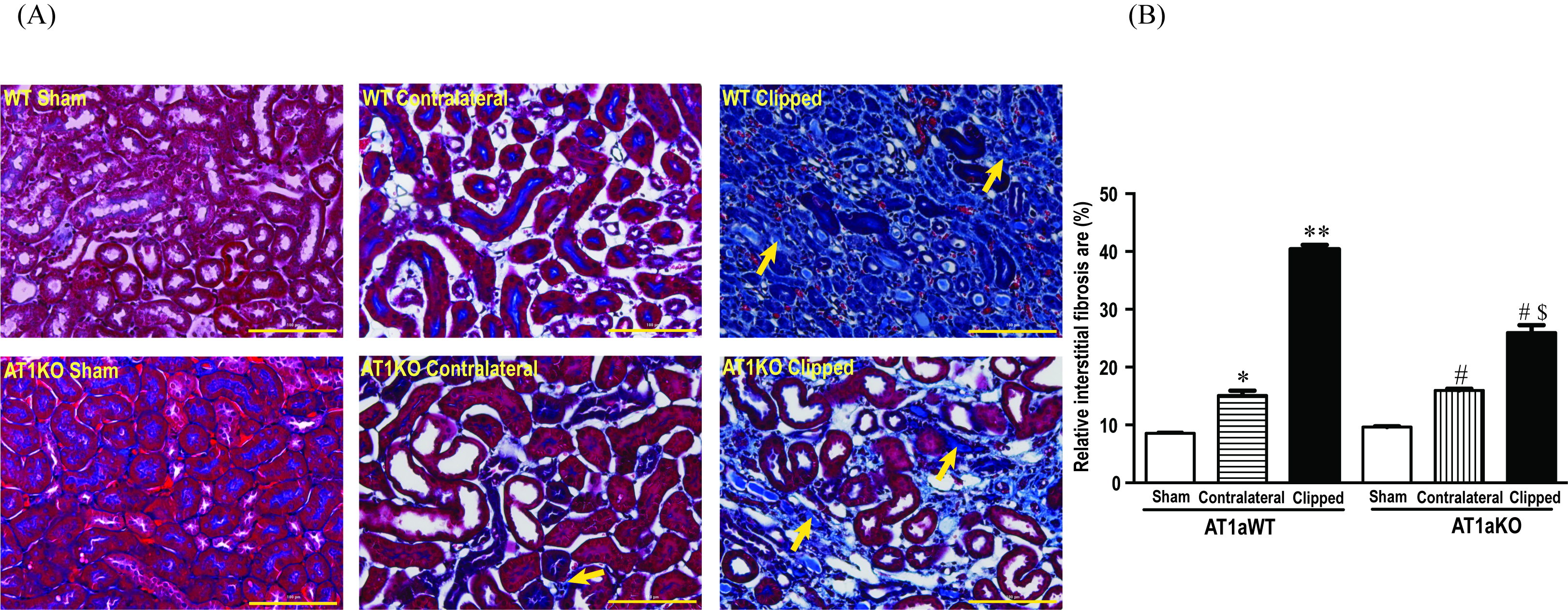

Supplement: Supplementary file 3 [file image2.tif]
